# Exploring fear in human-robot interaction: a scoping review of older adults’ experiences with social robots

**DOI:** 10.3389/frobt.2025.1626471

**Published:** 2025-10-13

**Authors:** Ahmed Elsheikh, Dena Al-Thani, Achraf Othman

**Affiliations:** 1 College of Science and Engineering, Hamad Bin Khalifa University, Doha, Qatar; 2 Mada Center, Doha, Qatar

**Keywords:** fear of robots, older adults, human-robot interaction, uncanny valley, trust, privacy, anxiety, social robotics

## Abstract

**Background:**

As global populations age, healthcare and social systems face mounting pressure to provide effective support for older adults. Social robots have emerged as promising tools to enhance companionship, cognitive engagement, and daily assistance. However, fear of robots among older adults remains a critical barrier to adoption.

**Objective:**

This scoping review examined how fear manifests in human-robot interaction (HRI), what factors contribute to these reactions, and how they influence technology acceptance.

**Methods:**

A systematic search of six major databases (PubMed, Scopus, IEEE Xplore, ACM Digital Library, PsycINFO, and Web of Science) identified studies published between January 2014 and March 2025. Following PRISMA-ScR guidelines, 49 studies were included, encompassing 6,670 older participants across 16 countries.

**Results:**

Thematic synthesis revealed seven main fear categories: privacy and autonomy concerns, trust and reliability issues, emotional and ethical discomfort, usability challenges, fear of dependence, unfamiliarity with technology, and the Uncanny Valley effect. Fear levels were shaped by robot design, cultural background, prior technology experience, and contextual factors such as care settings. Mitigation strategies, including co-design with older adults, gradual exposure, transparent system behavior, and emotionally congruent interaction, were associated with improved acceptance.

**Conclusions:**

This review uniquely maps fear typologies to robot functions and intervention strategies, offering a framework to guide emotionally adaptive and culturally sensitive robot design. Addressing emotional barriers is essential for the ethical and effective integration of social robots into eldercare. Future research should prioritize longitudinal, cross-cultural studies and standardized fear measurement tools to advance evidence-based HRI implementation.

## 1 Introduction

### 1.1 Background

Picture an elderly resident meeting a humanoid robot for the first time. The mixture of fascination and wariness in their reaction captures a fundamental dilemma confronting societies as they introduce robotic technologies into eldercare settings. With global demographics shifting dramatically, estimates indicate that by 2050, one-sixth of the world’s population will exceed 65 years of age (World Health Organization, 2022). Healthcare systems worldwide grapple with shrinking caregiver workforces and stretched resources. Social robots have gained recognition as valuable tools that can provide companionship, enhance cognitive functioning, and support daily activities (Chen and Song, 2019; Papadopoulos et al., 2020; Zafrani et al., 2023). These robotic solutions span from therapeutic animal-inspired designs, such as PARO, to advanced humanoid platforms created specifically for elderly care environments (Bemelmans et al., 2012; Broadbent et al., 2009). Technology offers reliable care delivery, individualized interaction, and lighter caregiver burdens, potentially addressing widespread social isolation among aging populations (Abdi et al., 2018; Robinson et al., 2014). Nevertheless, emotional barriers, fear being foremost among them, frequently obstruct widespread adoption and effective use. This fear reaches beyond simple technological unfamiliarity, touching on profound psychological, technological, and cultural concerns (Pu et al., 2019; Whelan et al., 2018). Fear can emerge as discomfort during robot-human exchanges, skepticism about robotic competence, or worries about personal autonomy and data protection (Imtiaz et al., 2024; Tobis et al., 2022). Considering the significant financial commitments being made in eldercare robotics, recognizing and addressing these fear-based obstacles becomes crucial for optimizing their impact and securing widespread acceptance among older populations (Chen, S. et al., 2018; Sawik et al., 2023).

### 1.2 Complexity of robot-related fear

Elderly individuals’ fearful reactions to eldercare robots involve intricate, overlapping factors. Seniors frequently experience anxiety that stems not merely from encountering unfamiliar technology, but from fundamental concerns about maintaining independence, protecting privacy, and preserving the human elements of care (Heerink et al., 2010; Naneva et al., 2020). Fear intensity varies considerably, spanning from subtle uneasiness to pronounced anxiety that leads to complete rejection of robotic interaction (Berns and Ashok, 2024; Olatunji et al., 2025). While younger people might regard robotic malfunctions as minor annoyances, elderly users view such errors, whether involving medication mistakes or inadequate emergency assistance, as serious threats to their safety (Nomura et al., 2005; Sharkey and Sharkey, 2012). Furthermore, Mori’s “Uncanny Valley” theory provides valuable insight into these fears of robots, explaining the discomfort that occurs when robots appear almost human but lack complete authenticity in appearance or behavior (MacDorman and Ishiguro, 2006; Miklósi et al., 2017; Mori, 1970). Consequently, developing emotionally appealing robots requires careful attention to human-like features to prevent triggering revulsion instead of promoting acceptance. Privacy anxieties add another layer of complexity to these fears. Elderly users often express concern about information misuse, constant monitoring, and diminished personal control when robots track health data or observe daily routines (Coco et al., 2018; Rantanen et al., 2018). Additionally, fears about becoming overly dependent on robotic support reflect broader concerns about aging processes, declining independence, and reduced human contact in caregiving (Baisch et al., 2018; Moyle et al., 2019).

### 1.3 Cultural and individual variations

Responses to social robots vary markedly across different personal backgrounds and cultural settings, underscoring the influence of social factors on technology interactions (Stafford et al., 2014; Torta et al., 2014). Previous technology experience reliably diminishes fear; elderly individuals who have used digital devices extensively show reduced anxiety and greater willingness to work with robotic caregivers (Nomura, T. et al., 2005; Strutz et al., 2024). Cultural background also plays a major role in shaping the fear of robots. Research reveals substantial differences between Eastern and Western perspectives, with East Asian populations, particularly in Japan and South Korea, typically showing less fear and greater acceptance than Western groups, mirroring broader societal views on automation and care practices (Backonja et al., 2018; Zhao et al., 2023). Western participants often focus more heavily on autonomy and privacy issues, aligning with cultural traditions that emphasize individual choice and the irreplaceable nature of human caregiving relationships (Carros et al., 2020; Zsiga et al., 2018). In addition, age-related differences within the elderly population also prove meaningful. Those in advanced age brackets (85+ years) may demonstrate different fear characteristics compared to younger seniors (65–74 years), possibly reflecting variations in technology exposure, cognitive adaptability, and health requirements (Conde et al., 2024; Yam et al., 2023). Gender distinctions have surfaced as well, with women generally focusing on emotional and interpersonal aspects, while men tend to emphasize practical and technical considerations (Jung et al., 2017; Leung et al., 2022). These patterns depend heavily on context and represent broader social influences rather than fundamental gender-based differences.

### 1.4 Evolving technological landscape

Recent developments in artificial intelligence, machine learning, and human-computer interaction have dramatically reshaped social robotics capabilities (Tay et al., 2014; Walters et al., 2008). Modern robots now incorporate sophisticated natural language processing, emotion detection, and behavioral adaptation, enabling more tailored and sensitive user interactions (Cavallo et al., 2018; Deutsch et al., 2019; Walters et al., 2008). Research methodologies have similarly progressed beyond simple self-reporting to include physiological measurements, behavioral analysis, and unconscious psychological evaluation techniques (Gasteiger et al., 2025; Koceski and Koceska, 2016). These approaches demonstrate that fear operates through both conscious and unconscious pathways, informing how interventions might better address these reactions (Takayanagi et al., 2014; Thunberg et al., 2022). Contemporary robot development emphasizes user-focused design principles, concentrating on emotional security, trust establishment, and gradual relationship building while maintaining functional excellence (Park et al., 2021; Sun and Ye, 2024). Empirical studies confirm that features such as motion naturalness, expressive interaction, and adaptive dialogue strongly influence user trust, acceptance, and fear responses (Fraune et al., 2020; Huang et al., 2024; Lubold et al., 2016; Yuan et al., 2024).

### 1.5 Rationale for this study

Although substantial resources have been invested in eldercare robotics, fear of robots remains poorly understood and inconsistently measured across the research literature, hampering practical applications. The scoping review provides a framework for thoroughly examining this varied and rapidly developing field. Unlike systematic reviews, scoping studies can incorporate diverse methodological approaches, theoretical frameworks, and research inquiries, effectively surveying broad knowledge bases in emerging areas such as human-robot interaction (Broadbent et al., 2009; Złotowski et al., 2015). This methodology enables the integration of quantitative, qualitative, and mixed-method investigations, creating a comprehensive understanding of current knowledge, pinpointing significant research limitations, and guiding future research priorities and implementation approaches (Laue et al., 2017; Tschöpe et al., 2017).

Moreover, although several systematic and scoping reviews, such as (Antona et al., 2019; Baisch et al., 2018; Tobis et al., 2022) have examined social robot use in eldercare, none provide a comprehensive synthesis focused on fear as a central emotional factor in technology acceptance and robot integration. Existing studies often mention fear indirectly or as part of broader acceptance measures, leaving their specific triggers and categories poorly defined. This scoping review addresses that gap by systematically examining empirical evidence on older adults’ fear of robots, including near-human (Uncanny Valley) discomfort, privacy and autonomy concerns, and dependence-related anxieties, across diverse interaction contexts and robot types. A key contribution of this review lies in its structured classification of fear types and their relationship to robot design features and user diversity, using systematic coding in NVivo to extract consistent thematic patterns. By also highlighting cross-cultural differences in emotional responses, the review underscores the need for localized and culturally sensitive design approaches. These contributions have direct practical value: they provide designers and engineers with evidence-based cues to improve user comfort, offer policymakers and health planners guidance for gradual and ethical deployment, and help care practitioners and families better prepare older adults for first encounters with social robots. By linking emotional barriers with actionable design and implementation strategies, this review bridges the gap between research insights and real-world application in eldercare robotics.

The remainder of this paper follows this organization: Section 3 outlines the study objectives and research questions. Section 4 outlines the methodological approaches following PRISMA-ScR standards. Section 5 reports synthesized findings and thematic categorizations. Section 6 relates results to existing scholarship, highlights knowledge deficits, and suggests future research pathways. Section 7 concludes with practical guidance for robot design and deployment, emphasizing psychological and emotional factors essential for elderly acceptance.

#### 1.5.1 Research questions

This investigation centers on three interconnected questions that emerged from our preliminary exploration of the literature and conversations with eldercare practitioners:

RQ1: What types of fear do older adults experience when interacting with social robots?

Instead of assuming fear is a uniform response, this question aimed to understand different ways fear and discomfort manifest when older adults interact with robots. This question explores both the obvious fears older adults readily describe, such as worries over their physical safety or privacy, and the more subtle, sometimes unconscious reactions that are shown through watching behavioral cues or taking physiological measures. The review is especially keen to see if different kinds of fears tend to group together or if they exhibit independently across different older adults and situations.

RQ2: What factors contribute to fear in older adults’ interactions with social robots?

Fear does not emerge spontaneously. This question examined the complex constellation of variables that shape older adults’ emotional responses to robotic systems. The investigation encompassed both observable characteristics, including robotic appearance and functional capabilities, and less apparent influences such as cultural contexts, prior technological encounters, and the social environments within which human-robot interaction occur. The study sought to identify the determinants of fear of robots and to understand how these diverse variables interconnect, potentially reinforcing or diminishing one another’s impact. Clarifying distinct fear categories helps caregivers and technology implementers with insights into customized interventions that can mitigate older adults’ fear.

RQ3: How does fear influence older adults’ acceptance and utilization of social robots?

The primary concern extends beyond types of fear manifestations to encompass their implications for technological adoption and sustained usage patterns. This investigation analyzed the mechanisms through which emotional responses are evident as behavioral outcomes, specifically, whether older adults completely avoid robotic systems, engage with them reluctantly, or develop strategies to surmount initial fears. The research focused particularly on determining whether fear reduction interventions could substantially enhance acceptance outcomes and identifying the underlying mechanisms that facilitate effective fear management.

## 2 Objectives

The principal objective was to construct a comprehensive synthesis of current knowledge regarding older adults’ fear of robots to social robots. This synthesis extended beyond mere cataloguing of existing studies to encompass understanding the landscape of research methodologies, participant demographics, and outcome measures employed in investigating fear of robots within human-robot interaction contexts. Through examination of this methodological diversity, the analysis aimed to identify both strengths and lacunae in contemporary research approaches. A secondary objective concentrated on elucidating how robotic characteristics influence emotional responses. Rather than conceptualizing robots as a homogeneous category, the investigation examined how specific design parameters encompassing appearance, movement patterns, interaction modalities, and intended functions shape fear of robots. This analysis provides evidence-based guidance for robot developers and implementers regarding design choices that may exacerbate or mitigate fear reactions. The third objective investigated the relationship between fear of robots and technology acceptance outcomes, exploring how emotional barriers influence older adults’ willingness to engage with robots and their patterns of actual usage. This analysis examined the potential for fear reduction interventions to improve acceptance and sustained engagement with social robots, while considering broader implications for eldercare technology implementation. Finally, the research aimed to present a conceptual framework that integrates findings across studies to illustrate relationships between fear types, contributing factors, mitigation strategies, and acceptance outcomes. This framework is designed to guide future research, inform intervention development, and support evidence-based decision-making in robot design and implementation. Rather than proposing a rigid theoretical model, the framework accommodates the complexity and variability observed in human-robot interaction while providing practical guidance for researchers and practitioners.

## 3 Methodology

### 3.1 Methodological framework and protocol development

The investigation was built on the methodological foundation established by (Arksey and O’malley, 2005), refined through Levac and colleagues’ subsequent improvements (Levac et al., 2010), and reported according to PRISMA-ScR guidelines (Tricco et al., 2018). This framework appealed to us because it provides structure for mapping complex, multidisciplinary topics while maintaining the flexibility essential for exploring emerging fields such as human-robot interaction in eldercare. In addition, the approach reflected a pragmatic approach, recognizing that understanding fear of robots in human-robot interaction requires drawing insights from gerontology, psychology, human-computer interaction, engineering, and healthcare (Johnson and Onwuegbuzie, 2004; Plano Clark, 2017). Rather than privileging any single disciplinary perspective, the study aimed to capture the full breadth of relevant knowledge while maintaining methodological rigor. A comprehensive review protocol was developed prior to initiating the search process. Although the protocol was not formally registered, this decision reflects the current limitations of platforms like PROSPERO, which accept only systematic reviews and meta-analyses. To maintain transparency and ensure reproducibility, the full protocol has been included as Supplementary Material 1, PRISMA-ScR Checklist Item. Its development involved collaborative discussions among the research team to define eligibility criteria, refine search strategies, and select appropriate synthesis methods. A preliminary pilot test using a small subset of studies (n = 5) allowed for practical adjustments, helping the team identify and address potential issues ahead of the full review process.

### 3.2 Eligibility criteria

Inclusion criteria balanced alignment with research objectives while maintaining feasible scope boundaries. The focus centered on adults aged 65 and older, consistent with established gerontological conventions, while recognizing the considerable diversity within this demographic. Studies examining mixed-age samples qualified for inclusion only when they offered distinct analyses for elderly participants or concentrated specifically on this population. Defining fear-related phenomena presented unexpected challenges. An expansive approach captured the complete spectrum of emotional responses, incorporating studies that examined fear, anxiety, discomfort, or other negative reactions that older adults displayed during interactions with humanoid or social robots. The inconsistent terminology found across research necessitated this broad conceptual framework to prevent overlooking valuable evidence.

The review incorporated multiple environmental contexts, residential settings, care institutions, research laboratories, and community spaces, reflecting the varied circumstances where elderly individuals encounter robotic technologies. Empirical investigations across all methodological approaches received consideration, encompassing quantitative, qualitative, mixed methods designs, and individual case studies. Case studies were included because they provide rich details about personal experiences, offering value when investigating emotional and psychological responses like fear within emerging technological domains. The temporal scope encompassed publications from January 2014 through March 2025 to maintain contemporary relevance. This timeframe encompasses recent developments in social robotics while excluding obsolete technologies. Resource constraints limited the search to English-language publications, potentially omitting relevant research published in other languages. Table 1 presents a comprehensive overview of inclusion and exclusion criteria.

**TABLE 1:** Eligibility criteria used to determine study selection for this scoping review.

Criteria	Inclusion	Exclusion
Population	Studies focused on adults aged 65 and above or included a subgroup analysis of older adults	Studies included only participants younger than 65 years
Concept	Studies focused on fear, anxiety, discomfort, or negative emotional reactions in older adults interacting with humanoid/social robots	• Investigation of non-humanoid robots (e.g., industrial robots, military robots, AI systems without a robotic embodiment) • Do not focus on negative emotional responses • Studies that only examine positive emotions (e.g., trust, empathy, enjoyment, performance, navigation) • Studies focused only on technical aspects or general technology adoption without addressing psychological or emotional fear
Context	Any setting where older adults interact with robots (e.g., homes, care facilities, laboratories)	Studies do not involve direct interaction with robots
Study Design	Empirical research (qualitative and quantitative), case studies, mixed methods studies, experimental studies and pilot studies	Purely technical or engineering-focused without human participant data
Publication Type	Peer-reviewed journal articles, conference proceedings, and book chapters	Systematic reviews, scoping reviews, meta-analyses, theoretical/conceptual papers, opinion articles, abstracts, short papers, theses, and dissertations
Time Frame	Studies published between January 2014 and March 2025 include the most recent humanoid and social robots	Studies were published before 2014, and some of those lacked a clear publication date
Language	English-language studies for consistency in review and analysis	Studies published in non-English languages

### 3.3 Search strategy

Developing an effective search strategy requires balancing sensitivity with specificity to capture all relevant studies while avoiding irrelevant results. Database selection aimed at comprehensive coverage across relevant disciplines. PubMed provided access to biomedical and medical literature, while IEEE Xplore captured engineering and technology perspectives. The ACM Digital Library covers virtually every aspect of computing and information technology, including Human-Robot Interaction (HRI), PsycINFO offers psychological research, Web of Science provides multidisciplinary coverage, and Scopus was selected for its broad multidisciplinary coverage, enabling the retrieval of peer-reviewed articles, conference papers, and gray literature across health, engineering, and social sciences. Additional studies were identified by hand-searching the included reference lists and relevant review articles. The last search was conducted on 15 March 2025. The search term development process involved identifying three primary concept domains: aging population terminology, robotic technology descriptors, and psychological response indicators. Within each domain, multiple synonyms and related terms were identified through preliminary searches, consultation with subject matter experts, and examination of key papers in the field. For the aging population domain, terms include “older adult,” “elderly,” “senior,” “aged,” “geriatric,” and “aging population.” The robotics domain encompasses “robot,” “humanoid,” “social robot,” “assistive robot,” “companion robot,” “socially assistive robot,” and “human-robot interaction.” The psychological response domain included “fear,” “anxiety,” “discomfort,” “negative emotion,” “acceptance,” “rejection,” “attitude,” “perception,” “uncanny valley,” and “technophobia.” The full list of search terms, including British and American spelling variations, is provided in Table 2.

**TABLE 2:** Keywords used for the database search.

Category	Synonyms/Related terms
Population	“older adults”, “elderly”, “senior”, “ageing population”, “aging population”, “geriatric”, “older people”, “older individuals”, “older persons”, “senior citizens”
Robots	“humanoid robots”, “social robots”, “robotic assistants”, “care robots”, “socially assistive robots (SARs)”, “service robots”, “HRI (human-robot interaction)”, “elderly care robots”, “eldercare robots”, “geriatric robots”
Fear	“fear of robots”, “technology anxiety”, “technological anxiety”, “robot fear”, “robot-related apprehension”, “uncanny valley”, “distrust in robots”, “hesitation toward robots”, “discomfort with robots”, “fear of automation”, “technology-related distress”, “psychological distress during HRI”, “robot-induced stress”, “robot-related anxiety”
Context/Setting	“eldercare”, “elder care”, “geriatric healthcare”, “geriatric healthcare”, “long-term care”, “aging services”, “ageing services”, “nursing homes”, “residential homes”, “independent living communities”, “healthcare”, “healthcare”, “assisted living”, “home care”, “domiciliary care”, “care homes”, “senior living communities”

Boolean operators (AND, OR) were employed to combine terms within and across concept domains, adapting the strategy for each database while maintaining conceptual consistency. Truncation and wildcard symbols facilitated the capture of variations in terminology. Beyond database searching, the investigation included hand-searching the reference lists of included studies and relevant review articles, as well as conducting citation tracking for key papers to identify more recent work. The detailed search strategy, including full Boolean strings and field specifications for each electronic database, is documented in Supplementary Appendix A.

### 3.4 Selection process

**FIGURE 1:**
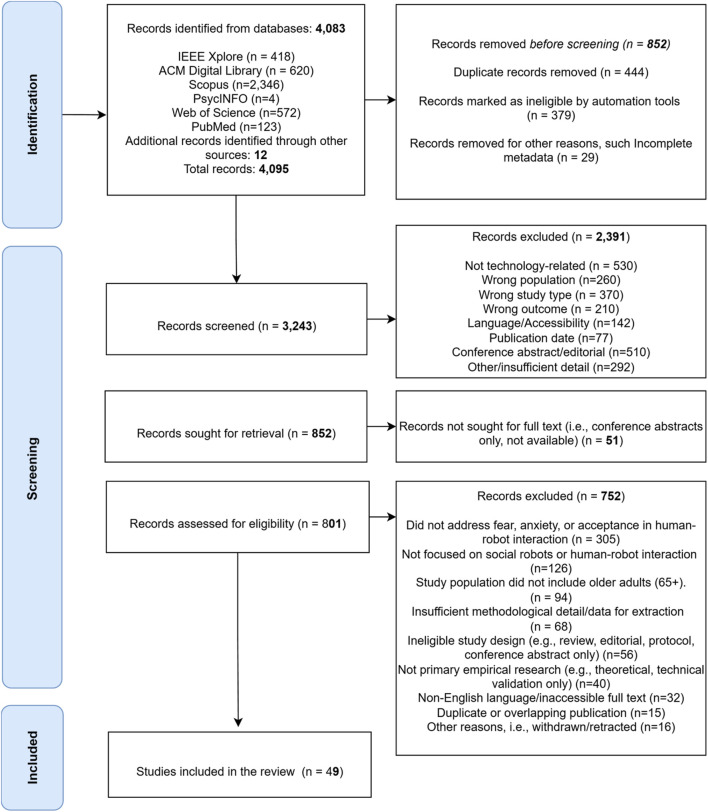
PRISMA-ScR (Tricco et al., 2018) flow diagram showing the study selection process with detailed exclusion reasons at each stage.

Studies published from January 2014 to March 2025 were considered, aligning with the period of significant advancement in social and humanoid robotics relevant to eldercare (Goeldner et al., 2015). The review adhered to PRISMA-ScR guidelines (Tricco et al., 2018) to ensure methodological rigor and transparency. After duplicate removal in RefWorks, two reviewers independently screened titles and abstracts in Rayyan (Ouzzani et al., 2016) using predefined inclusion/exclusion criteria. Full texts of potentially eligible articles were then reviewed by both reviewers. Discrepancies were resolved by discussion or, when necessary, by a third reviewer. Cohen’s kappa coefficients indicated substantial agreement at both screening stages (title/abstract κ = 0.78; full text κ = 0.85). The database searches yielded a total of 4,083 records: PubMed (n = 123), IEEE Xplore (n = 418), ACM Digital Library (n = 620), PsycINFO (n = 4), Scopus (n = 2,346), and Web of Science (n = 572). An additional 12 studies were identified by hand-searching the reference lists of included articles, ensuring comprehensive coverage. Following duplicate removal and application of eligibility criteria, 49 studies were included in the final synthesis. The full selection process is illustrated in Figure 1 (PRISMA-ScR flow diagram).

While this time frame ensures contemporary relevance, the exclusion of studies published before 2014 may omit foundational theoretical work in human-robot interaction. This limitation is further addressed in the Discussion section.

### 3.5 Data charting process

A standardized data extraction form was developed and piloted with five randomly selected studies: (Baisch et al., 2018; Chen and Song, 2019; Nault et al., 2024; Robinson et al., 2014; Ostrowski et al., 2019). These studies were selected to represent diversity in study design, robot type, and outcome measures. The pilot process revealed the need for additional fields related to fear assessment methods and mitigation strategies, leading to the refinement of the extraction form. Further, data extraction was then performed comprehensively across all included studies using the finalized form to ensure methodological rigor, reliability, and clarity. For each study, two reviewers independently extracted data, focusing on all elements critical to subsequent analysis and synthesis. Discrepancies were resolved by discussion and consensus.

The core elements of the extracted data are summarized in Table 3, and the complete extraction form is provided as Supplementary Material 2. Consistent application of the standardized extraction process was maintained throughout the review.

**TABLE 3:** Data extraction elements and descriptions.

Element	Description
Author(s)	To credit the original work
Year of Publication	To assess the recency and relevance of the findings
Geographical Location	To understand cultural contexts influencing the fear of robots
Study Design	To categorize research methodology (qualitative, quantitative, mixed methods)
Participant Demographics	Age, gender, health status, and cultural background (to understand the study population)
Type of Robot	Robot characteristics (physical/virtual, humanoid/non-humanoid) are used to understand the influence of design on fear
Main Findings (Fear)	Specific results on older adults’ fear, anxiety, or acceptance of robots
Interventions (Fear)	Details and outcomes of interventions used to reduce fear
Key Findings Related to Fear of Robots	Main results related to the research questions

The full synthesis of extracted studies, including methodological approaches, key findings, and fear mitigation interventions, is provided in Supplementary Appendix Table 6. This comprehensive overview supports evidence based on the fear of robots among older adults interacting with humanoid robots.

### 3.6 Quality assessment: evaluating diverse study designs

**FIGURE 2:**
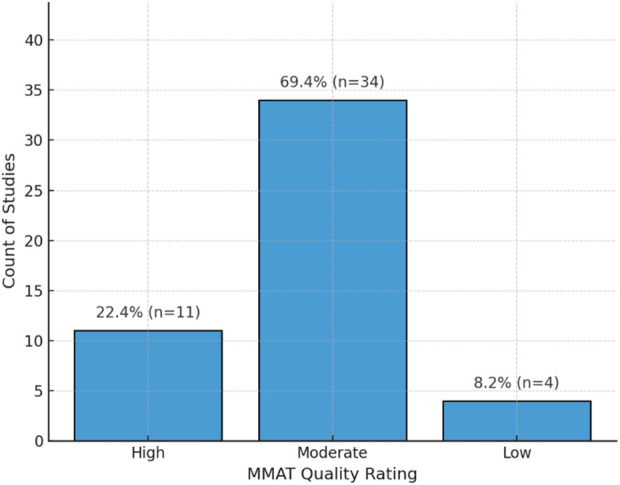
Distribution of the included studies by methodological quality, assessed using the Mixed Methods Appraisal Tool (MMAT). Most studies were rated as moderate quality, with fewer achieving high or low ratings.

The assessment of research rigor across incorporated investigations encountered distinctive obstacles stemming from varied experimental approaches. The Mixed Methods Appraisal Tool (MMAT), 2018 edition (Hong et al., 2018) served as the evaluation instrument, offering systematic examination capabilities for quantitative, qualitative, and combined methodological frameworks through a cohesive structure. Two independent assessors examined each investigation against design-appropriate MMAT standards, with consensus achieved through collaborative discussion for any initial disparities. The MMAT generates proportional scores (0%–100%) that reflect adherence to established quality benchmarks, allowing for cross-design comparisons. Among the 49 incorporated investigations, 11 (22.4%) were rated as high quality (80%–100%), 34 (69.4%) were assessed as moderate quality (60%–79%), and 4 (8.2%) were found to have lower methodological rigor (below 60%). These results are visualized in Figure 2, highlighting the predominance of moderate-quality studies, a reflection of both the emerging nature of the field and the practical difficulties associated with conducting rigorous empirical research in human-robot interaction involving older adults. The comprehensive methodological evaluation outcomes for all investigations are presented in Supplementary Material 3. Moreover, while all studies were retained in the synthesis regardless of quality rating, greater interpretative weight was assigned to findings from higher-quality studies. This approach ensures that conclusions are grounded in robust evidence while preserving a comprehensive view of the available literature.

### 3.7 Synthesis of results

**FIGURE 3:**
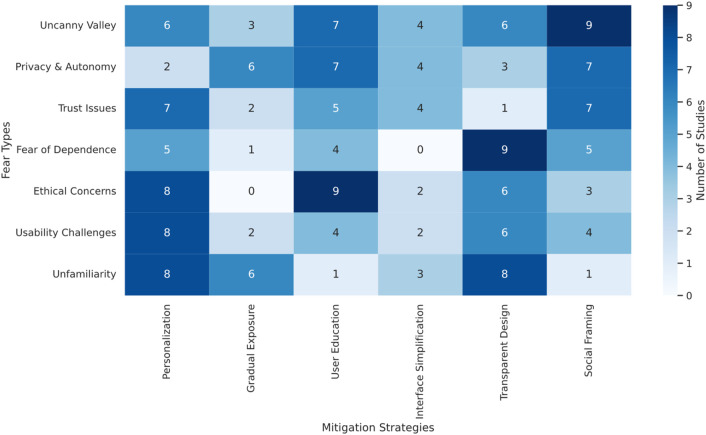
Heatmap showing how different fear types align with mitigation strategies across 49 studies. Darker shades indicate stronger evidence of association. Numbers indicate the count of studies (see Supplementary Appendix C for full mapping of each cell to specific studies). Example: The “6” in the Uncanny Valley × Personalization cell corresponds to Appel et al. (2019), Berns and Ashok (2024), Mishra et al. (2022), Strutz et al. (2024), Dosso et al. (2023), and Yam et al. (2023).

The methodological heterogeneity across incorporated investigations rendered meta-analytical approaches impractical. Consequently, descriptive synthesis integrated with thematic examination was implemented. Data management and analysis utilized NVivo 14.0, incorporating both inductive and deductive coding methodologies. Deductive themes were drawn from established theoretical foundations, including the Uncanny Valley hypothesis (Mori, 1970), technology acceptance frameworks (Davis et al., 1989; Venkatesh et al., 2003), and psychological models of trust and anxiety in human-machine interaction (Nomura et al., 2006). Simultaneously, the coding protocol accommodated emergent, data-derived themes. Two investigators conducted an independent study review and coding, convening regularly to address discrepancies and refine the developing thematic architecture. This cyclical process ensured analytical consistency while capturing both anticipated and unanticipated insights. The final synthesis matrix (Figure 3) was developed through iterative thematic coding and frequency analysis, capturing the association between specific fear types and mitigation strategies across the included studies. This heatmap visually conveys the strength of these associations, highlighting patterns of co-occurrence within the dataset. Dark cells indicate a higher number of studies reporting the linkage between a given fear and the corresponding mitigation approach that emerged through successive analytical phases, commencing with theory-informed structure and evolving in response to observed data patterns. To ensure transparency, each numerical value shown in Figure 3 is mapped to the exact study references in Supplementary Appendix C. This model illustrates the interconnections among fear of robots categories, influencing variables, mitigation approaches, and outcomes pertinent to research objectives. To enhance synthesis credibility, methodological quality assessment for each study employed the Mixed Methods Appraisal Tool (MMAT, version 2018) (Hong et al., 2018). Finding interpretation prioritized studies rated as high quality or of adequate quality strengthened the reliability of the synthesized themes.

The synthesis process was guided by research questions, with particular attention to ensuring that findings related to fear factors (RQ2) and acceptance relationships (RQ3) were analyzed and presented with the same depth and rigor as those related to fear types (RQ1). Multiple analytical approaches were employed to address each research question comprehensively.

## 4 Results

### 4.1 Study selection and characteristics

The systematic search yielded 4,095 records across six databases and supplementary sources. During pre-screening, 852 records were excluded, 444 as duplicates, 379 via automated filters, and 29 for incomplete metadata or non-research formats. The remaining 3,243 records underwent title and abstract screening, leading to the exclusion of 2,391 articles based on the following criteria: non-technological focus (n = 530), irrelevant populations (n = 260), unsuitable study types (n = 370), outcomes unrelated to fear or acceptance (n = 210), language or access limitations (n = 142), out-of-range publication years (n = 77), non-peer-reviewed content (e.g., editorials, abstracts; n = 510), and insufficient methodological information (n = 292). Of the 852 full texts sought, 51 were unavailable or deemed out of scope, leaving 801 for detailed review. A further 752 were excluded for reasons including: absence of fear, anxiety, or acceptance focus (n = 305), lack of emphasis on social or humanoid robots (n = 126), exclusion of older adults (65+) as a study population (n = 94), insufficient methodological clarity (n = 68), ineligible design (n = 56), non-empirical format (n = 40), language/inaccessibility issues (n = 32), duplication (n = 15), and withdrawal or retraction (n = 16). The total of 49 studies met all inclusion criteria and were included in the final synthesis (Figure 1).

### 4.2 Publication years and trends

**FIGURE 4:**
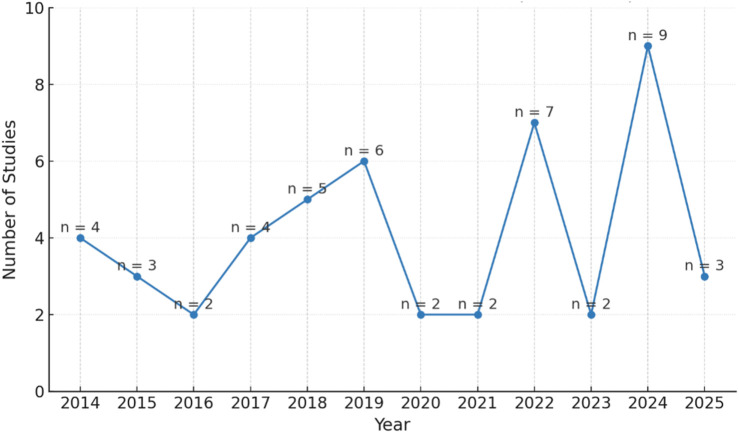
Temporal distribution of the 49 included studies (2014–March 2025), with publication peaks observed in 2021 and 2024, indicating rising scholarly attention to fear of robots in older adults during human-robot interaction.

The temporal distribution of included studies (Figure 4) highlights a progressive increase in research activity addressing fear-related responses among older adults toward social robots between January 2014 and March 2025. This upward trajectory indicates sustained academic interest in understanding the emotional and psychological dimensions of human-robot interaction in aging populations. Notably, the years 2021 and 2024 recorded the highest volume of publications, with nine studies each, underscoring a surge in empirical focus during these periods. The trend reflects growing recognition within the research community that fear represents a substantive barrier to the adoption of robotic technologies in eldercare contexts. As such, the data point toward an urgent need for age-sensitive design approaches and targeted intervention strategies that address emotional safety and user trust.

### 4.3 Sample characteristics

**FIGURE 5:**
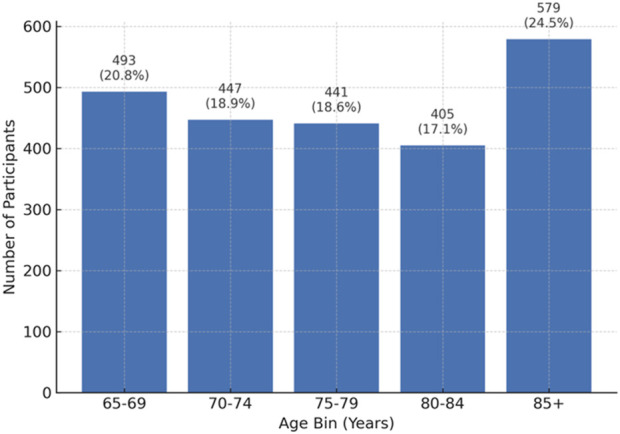
Age Distribution of Participants in 49 Included Studies (n = 6,670). The largest proportion of participants were 85+ years (24.5%), reflecting a focus on very old adults in studies of human-robot interaction.

The 49 included studies involved a total of 6,670 older adult participants, with individual study sizes ranging from 12 to 384 (mean = 136; median = 67). Figure 5 shows the age distribution of participants. The largest proportion was 85 years and older (24.5%), followed by 65–69 years (20.8%), 70–74 years (18.9%), 75–79 years (18.6%), and 80–84 years (17.1%). This pattern reflects the field’s growing focus on very old adults, who represent the population most likely to interact with assistive and socially engaging robotic technologies. By including a substantial number of participants aged 85 years and above, recent studies capture the experiences of individuals who are both most in need of and most sensitive to the design, emotional safety, and usability of social robots.

### 4.4 Methodological approaches

**FIGURE 6:**
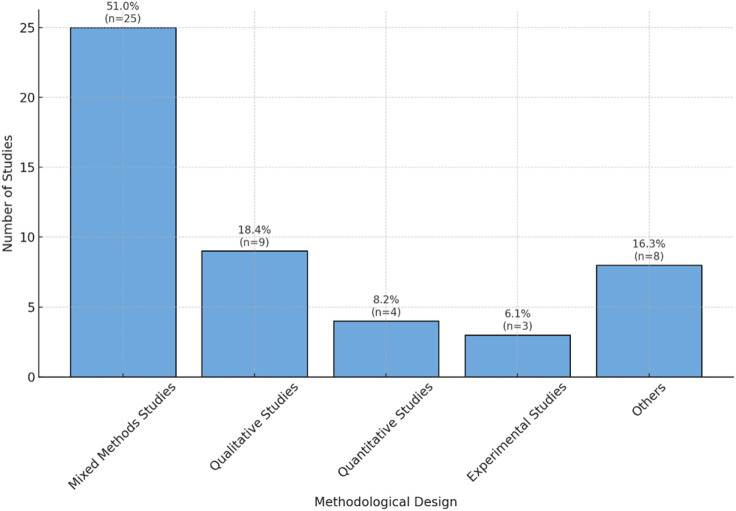
Distribution of methodological approaches across the included studies, showing the frequency of quantitative, qualitative, mixed-methods, and other designs used to explore fear in older adults’ interactions with robots.

Reviewed studies demonstrated considerable methodological variation. As illustrated in Figure 6, mixed-method designs dominated the literature, comprising 51.0% (25 studies). Qualitative approaches ranked second at 18.4% (9 studies), while exclusively quantitative methods constituted 8.2% (4 studies). Experimental designs accounted for 6.1% (3 studies), with additional methodologies including comparative analyses, cross-sectional surveys, and narrative reviews, each representing roughly 2.0%. This methodological heterogeneity reflects the intricate challenges involved in examining emotional responses among older adults during robotic interactions.

### 4.5 Robotic platforms and assessment tools

The robotic systems examined in our analysis demonstrated considerable heterogeneity. Among the platforms investigated most frequently were Pepper (n = 2, 4.1%), Jibo (n = 2, 4.1%), PARO (n = 2, 4.1%), NAO (n = 2, 4.1%), and Kompaï (n = 2, 4.1%). Such diversity reflects both the dynamic development within social robotics research and reveals an absence of consistent methodological standards across contemporary investigations. Prior work highlights that differences in platform type can also shape perceived competence, engagement, and comfort in human-robot interaction (Görer et al., 2017; Harrison, 2015; Spatola et al., 2021). Furthermore, researchers employed varied approaches when assessing fear and anxiety responses, incorporating established psychometric instruments alongside purpose-built questionnaires and behavioral observation techniques.

### 4.6 Functional classification of robotic systems

**FIGURE 7:**
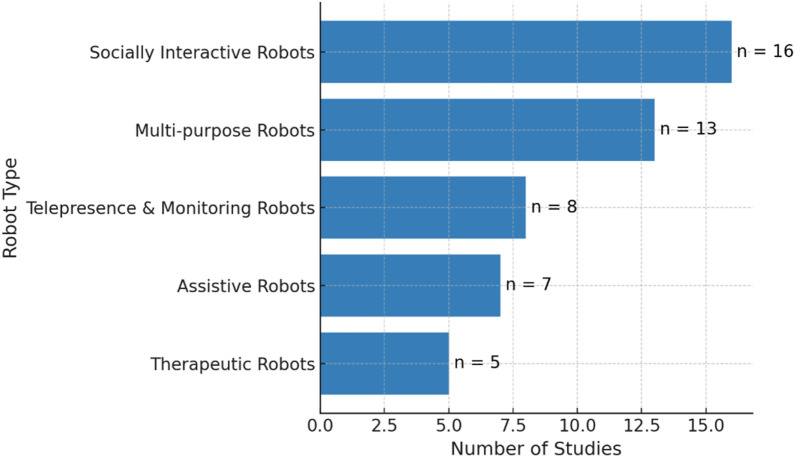
Functional classification of robots used in the 49 included studies, with socially interactive and multi-purpose robots being the most frequently investigated types.

The robotic technologies examined across the literature were categorized into five functional groups according to their predominant roles in geriatric care environments. Robots designed for assistance (7 studies, 14.3%), such as RAMCIP and Robot-Era platforms, were developed to help elderly individuals with routine activities, including movement support, medication scheduling, and personal hygiene tasks. Those serving therapeutic purposes (5 studies, 10.2%), notably Paro and Telenoid systems, concentrate on delivering emotional support and cognitive enhancement or facilitating physical recovery through purposefully designed interactive experiences. Platforms focused on social engagement (16 studies, 32.7%), including Pepper and NAO units, were primarily created to offer companionship while mitigating isolation and encouraging interpersonal connections among aging populations. Communication and surveillance systems (8 studies, 16.3%), exemplified by the Giraff platform, enable distant correspondence, medical assistance, and environmental observation, establishing essential links between seniors and both healthcare providers and relatives across geographic distances. Finally, integrated systems (13 studies, 26.5%) combine multiple functions, incorporating companion services, practical assistance, and supervisory capabilities for deployment in private residences or institutional care facilities. This category encompasses devices such as RobuLAB 10, LOVOT, and Ubtech Alpha Mini. The distribution patterns shown in Figure 7 reveal that contemporary research demonstrates marked interest in robots capable of social engagement and those offering combined functionalities.

### 4.7 Fear assessment tools

**FIGURE 8:**
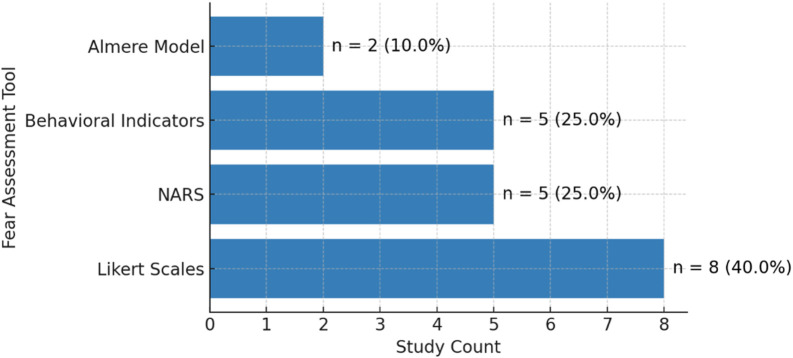
Frequency of fear assessment tools used across the 49 included studies, showing the predominance of standardized scales such as NARS, and the Almere Model.

There was notable variation in how the included studies assessed fear and emotional responses during human-robot interaction. The most frequently employed tool was the Likert scale (Likert, 1932), which was used in eight studies to quantify attitudes, comfort, and perceptions of robotic systems. Both the Negative Attitudes Toward Robots Scale (NARS) (Nomura, T. et al., 2005) and behavioral observation methods, such as video-based coding or documented reactions, were utilized in five studies. The Almere Model (Heerink et al., 2010) for technology acceptance, was reported in two studies. Other approaches, including open-ended interviews and thematic analysis, contributed further qualitative insight. However, no single standardized anxiety scale, such as the State-Trait Anxiety Inventory (Spielberger and Gorsuch, 1983) was identified in this sample. This diversity in assessment strategies highlights the field’s reliance on both structured quantitative instruments and behavioral or narrative methods to capture the range of fear and acceptance responses among older adults. Figure 8 summarizes the distribution of the main assessment tools used across all studies.

### 4.8 Thematic analysis: origins and types of fear

The thematic analysis in this scoping review aimed to identify, categorize, and contextualize fear-related responses of older adults interacting with robots. A detailed thematic analysis was conducted across the full texts of the 49 included studies to systematically investigate the origins and expressions of fear experienced by older adults during interactions with social robots. Each study was independently coded by two researchers, with discrepancies resolved through consensus discussions. NVivo tools were used to conduct matrix coding queries and visualize frequency distributions and co-occurrence of fear types, robot categories, and participant variables. Employing NVivo 14.0, a rigorous, multi-stage approach was implemented that blended inductive and deductive logic. This allowed both emergent and theory-driven themes to be captured and analyzed systematically. This dual approach allowed overt and subtle indicators of fear to be identified, ranging from explicit anxiety or avoidance to less immediately visible concerns, such as privacy, ethical discomfort, or feelings of dependence. Thematic coding began with a comprehensive word frequency analysis, focusing on qualitative data from all studies (Supplementary Material 4: Word Cloud illustrations). After standard preprocessing (stop word removal, stemming, and phrase grouping), common terms such as “fear,” “privacy,” “trust,” and “robot” emerged as highly salient. However, to move beyond mere frequency counts, themes were organized into seven principal categories based on both coding cycles and co-occurrence across robot types and user age groups. These emergent themes are summarized in Table 4. These encompassed the Uncanny Valley phenomenon, privacy and autonomy concerns, trust and reliability issues, dependence-related fears, emotional and ethical discomfort, usability obstacles, and insufficient prior technological exposure. The prevalence of these themes fluctuated according to both robotic morphology and participant demographics. For instance, participants aged 76–85 most reported Uncanny Valley phenomena discomfort or aversion elicited by lifelike yet subtly artificial humanoid platforms, such as NAO and Pepper. Conversely, privacy and autonomy concerns predominated among participants aged 81 and above, particularly during interactions with surveillance and remote presence systems, where users frequently expressed anxiety regarding observation and diminished personal control. Significantly, the youngest cohort (65–70 years) demonstrated a greater likelihood of experiencing fear due to technological unfamiliarity, though this was often ameliorated through structured exposure and supportive introduction protocols.

**TABLE 4:** Themes of fear identified across 49 studies. Percentages are relative to the total number of included studies. Representative robot types, cohorts, and key fear characteristics are shown.

Theme	% studies (of 49)	Common robot types	Typical cohorts (yrs)	Key characteristics (with references)
Trust and Reliability	20 (40.8%)	NAO, Pepper, Jibo, and other socially assistive robots	65–95	Concerns about dependability, safety, and system breakdowns in daily living and healthcare. Documented in: Baisch et al., 2017; Dosso et al., 2023; Giorgi et al., 2022; Ostrowski et al., 2019; Ostrowski et al., 2024; Strutz et al., 2024; Tobis et al., 2022; Wu et al., 2014; Yam et al., 2023; Zafrani et al., 2023; Coco et al., 2018
Privacy and Autonomy Concerns	14 (28.6%)	Telepresence robots, AI-driven systems, and Pepper	60–99	Fears of surveillance, data misuse, and diminished personal agency, particularly in care and monitoring contexts. Reported by: Coco et al., 2018; Rantanen et al., 2018; Søraa et al., 2022; Rigaud et al., 2024; Zsiga et al., 2018; Zafrani et al., 2022; Yam et al., 2023
Emotional and Ethical Concerns	14 (28.6%)	PARO, Pepper, Sil-Bot, NAO	65–95	Worries about emotional deception, infantilization, or manipulation of vulnerable users. Supported by: Jung et al., 2017; Moyle et al., 2019; Vandemeulebroucke et al., 2019; Sharkey and Sharkey, 2012; Vozna andand Costantini, 2025; Coco et al., 2018; Søraa et al., 2022; Rigaud et al., 2024; Zafrani et al., 2023
Lack of Prior Exposure	8 (16.3%)	Pepper, NAO, general-purpose robots	54–98	Anxiety arising from unfamiliarity with robots, often alleviated after direct interaction or repeated use. Documented in: Baisch et al., 2017; Carros et al., 2020; Cavallo et al., 2018; Gasteiger et al., 2025; Nault et al., 2024; Olatunji et al., 2025; Ostrowski et al., 2019; Strutz et al., 2024
Usability Challenges	5 (10.2%)	Sil-Bot, CO-HUMANICS, Robot-Era	65–86	Reluctance due to technical complexity, interface difficulties, or poor accessibility. Found in: Carros et al., 2020; Cavallo et al., 2018; Gasteiger et al., 2025; Nault et al., 2024; Olatunji et al., 2025; Strutz et al., 2024; Wu et al., 2014
Uncanny Valley Effect	4 (8.2%)	Humanoid robots: Pepper, NAO MAH, ROMAN, ROBIN, androids, Ethorobots, Ellix	50–85	Unease with human-like appearance or unnatural movement, leading to discomfort and avoidance. Reported in: Appel et al., 2019; Berns and Ashok, 2024; Mishra et al., 2022; Strutz et al., 2024; Dosso et al., 2023; Yam et al., 2023; Złotowski et al., 2015; Yamaguchi, 2025; Zafrani et al., 2023
Fear of Dependence	3 (6.1%)	Aldebaran NAO, Kompaï, TIAGo	65–94	Anxiety over reduced human contact or overreliance on robotic assistance. Reported in: Baisch et al., 2017; Dosso et al., 2023; Ostrowski et al., 2019; Ostrowski et al., 2024; Tobis et al., 2022; Wu et al., 2014; Zsiga et al., 2018; Rigaud et al., 2024; Zafrani et al., 2023

**FIGURE 9:**
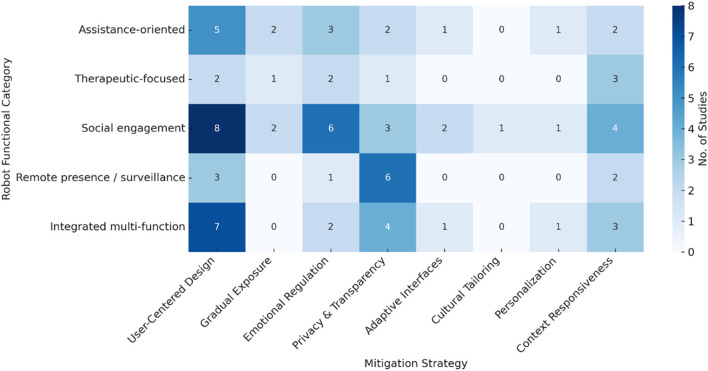
Heatmap showing the number of studies linking robot categories to fear-reduction strategy domains. Numbers indicate the count of studies; see Supplementary Appendix D for the full mapping. Example: The “8” for Social Engagement Robots × User-Centered Design corresponds to Carros et al. (2020), Søraa et al. (2022), Ostrowski et al. (2019), Ostrowski et al. (2024), Strutz et al. (2024), Nault et al. (2024), Zafrani et al. (2023), and Yam et al. (2023).

**FIGURE 10:**
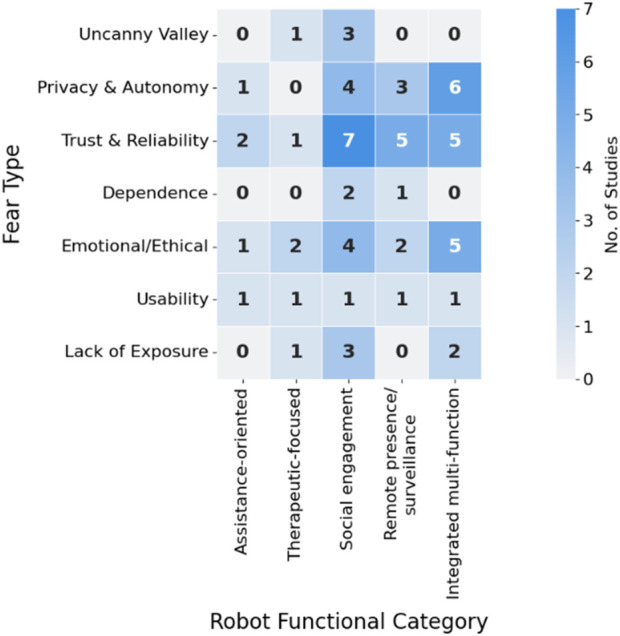
Heatmap summarizing how mitigation strategies are distributed across different functional categories of robots. The numbers indicate how many studies reported each link. Full reference lists for the studies represented in each cell are provided in Supplementary Appendix E. For example, the value “7” in the cell for Integrated Multi-function Robots × User-Centered Design corresponds to Rigaud et al. (2024), Zsiga et al. (2018), Carros et al. (2020), Ostrowski et al. (2024), Nault et al. (2024), Olatunji et al. (2025), and Gasteiger et al. (2025).

Eight different approaches to reducing fear were identified across the reviewed studies. Some of these were documented repeatedly, while others appeared only once or twice. The strategies most often described were participatory or co-design methods (n = 8), the use of emotional expressions such as affective speech or gestures (n = 6), and features that emphasized transparency and privacy (n = 6). Other techniques were far less common: gradual exposure protocols (n = 2), adaptive interface adjustments (n = 2), cultural tailoring (n = 1), personalization (n = 1), and context-responsive interactions (n = 3). The uneven distribution of these practices shows that current work is still exploratory, with little replication and limited consensus on best practice. In addition, the distribution of strategies was closely tied to the function of the robots themselves and the situations in which they were introduced. Social engagement robots were most often linked with participatory design and emotionally supportive interactions, which fit their role in companionship and social contact. Assistance-oriented and therapeutic robots showed more moderate use of emotional regulation and gradual exposure, reflecting their deployment in supportive or rehabilitative contexts. In contrast, remote presence and integrated multi-function robots were largely associated with transparency and privacy-related measures, along with some participatory elements. Taken together, the evidence suggests that mitigation strategies are applied across all categories of robots, but with considerable variation. For transparency, Figures 9, 10 show only the counts, while the detailed study-by-study mapping is provided in Supplementary Appendixs D, E.

Figure 10 provides an overview of how mitigation strategies are distributed across different categories of robots used in eldercare. Five main groups are represented—assistance-oriented, therapeutic, socially interactive, remote presence, and integrated multi-function platforms—set against four domains of intervention: emotional regulation, participatory or user-centered design, privacy and autonomy safeguards, and context-sensitive interaction. Patterns varied across robot types. Social and therapeutic robots were often associated with discomfort linked to human-like appearance and emotional unease. In these cases, design choices that emphasized user involvement and emotionally supportive interaction were the most frequently reported strategies. By contrast, concerns over surveillance, loss of control, and data handling were more often raised in relation to remote presence and multifunctional systems, where transparency and explicit user control measures were seen as central. Assistance-oriented devices drew on a combination of participatory design, simplified interfaces, and privacy safeguards to address similar issues. In addition, the distribution of strategies also differed by user group. Older participants expressed stronger reactions to uncanny valley effects and emotional discomfort, whereas younger and more technologically familiar cohorts showed lower levels of fear and engaged more readily with the devices. As the heatmap indicates, socially interactive and therapeutic robots were more frequently linked with user-centered and emotional regulation approaches, while remote and assistive systems tended to emphasize privacy protections and usability. These differences underline the importance of tailoring fear-reduction measures not only for the functional purpose of the robot but also to the characteristics and expectations of the people interacting with it. Full details of the study mappings that underpin these patterns are available in Supplementary Appendix E.

## 5 Discussion

This scoping review highlights the complex and multidimensional nature of fear of robots among older adults interacting with social robotic systems. As populations age globally, understanding and mitigating these emotional responses is critical to the responsible integration of robotic technologies in geriatric care. The discussion situates the findings within key theoretical frameworks, including the Uncanny Valley Hypothesis (Mori et al., 2012) and the Technology Acceptance Model (TAM), and examines emerging patterns across demographic, cultural, and robot design factors. Table 5 summarizes the study’s three guiding research questions (RQs), the main thematic findings, illustrative insights drawn from each theme, and remaining gaps identified in the literature.

**TABLE 5:** Central research questions (RQs) thematic results.

Research question	Key themes	Key insights	Identified gaps
RQ1: What types of fear do older adults experience when interacting with social robots?	• Anticipatory anxiety • Uncanny Valley responses • Loss of control • Functional distrust	• Fear arises from uncertainty about robot behavior and perceived autonomy. • Aesthetic discomfort is most notable with humanoid robots. • Concerns include dependence and privacy	• No standardized classification of fear types. • Fear is often measured indirectly rather than as a central construct
RQ2: What factors contribute to fear in older adults’ interactions with social robots?	• Media influence and fictional narratives • Prior negative technology experiences • Social influence and peer opinion • Generational technological gap	• Media portrayals create negative expectations. • Past technology failures foster skepticism. • Peer and caregiver influence can reduce fear	• Few studies address the socio-cultural origins of fear. • No standard tool to distinguish contextual from internalized fear
RQ3: How does fear influence older adults’ acceptance and utilization of social robots?	• Emotional barriers to engagement • Perceived usefulness vs. emotional discomfort • Trust calibration	• Fear decreases acceptance even when utility is recognized. • Trust and familiarity can reduce fear over time	• Lack of longitudinal studies examining fear and acceptance trajectories. • Few proactive interventions target initial fear reduction

### 5.1 Types of fear in human-robot interaction (RQ1)

Older adults’ fear of robots during interactions with social robots typically falls into four primary categories: anticipatory anxiety, uncanny valley effects, perceived loss of autonomy, and functional distrust. These categories collectively shape both emotional and behavioral reactions in human-robot encounters. Anticipatory fear stems from uncertainty about the robot’s intentions or next actions. For example, (Lima et al., 2022), reported that older users expressed anxiety when robots acted unpredictably or failed to communicate with a clear intent. Uncanny valley reactions, based on the well-established framework by Mori, (1970) and later expanded by (Macdorman and Minato, 1970; Mori et al., 2012), describe discomfort caused by humanoid robots that appear nearly, but not fully, human. Studies such as (Mishra et al., 2022; Tulsulkar et al., 2021) noted that elderly participants reacted negatively to robots exhibiting near-human traits like blinking, gesturing, or artificial voice, which reduced willingness to engage. Comparable findings demonstrate that robot appearance, movement quality, and social presence cues are central to triggering or alleviating fear in older adults (Fraune et al., 2020; Görer et al., 2017; Huang et al., 2024; Spatola et al., 2021; Yuan et al., 2024). Concerns around autonomy and privacy were particularly salient in healthcare contexts (Søraa et al., 2022). found that anxiety increased when robots collected sensitive information or operated independently. Similarly, (Dosso et al., 2023), observed that dependency on robots for essential tasks like medication reminders or mobility support raised fears of emotional distancing and reduced human oversight.

Functional skepticism, or doubts about the robot’s reliability, was another key theme (Ostrowski et al., 2019). highlighted that older adults feared malfunctions or inappropriate responses from robotic caregivers, potentially endangering safety or diminishing human involvement. Despite these consistent observations, a significant methodological limitation persists, while some investigations explicitly measured fear using structured instruments (Appel et al., 2019; Macdorman and Minato, 1970; Mori, M. et al., 2012; Pino et al., 2015), most inferred fear indirectly, utilizing behavioral withdrawal, qualitative indicators, or broader attitude scales such as NARS (Nomura et al., 2006) and the Almere Model (Heerink et al., 2010). Consequently, the absence of a standardized framework for categorizing and measuring fear types in human-machine interaction with elderly populations constrains the capacity to conduct comparative analyses or develop targeted interventions.

### 5.2 Origins of fear: internal and external influences (RQ2)

The fear of robots toward social robotic platforms is influenced not solely by the platforms’ physical appearance or behavior but also by deeper psychological and socio-cultural elements. Four primary origins were identified: media influence and fictional narratives, previous adverse technology experiences, social and peer influence, and the generational digital divide (see Table 5). Media narratives and fictional portrayals exert substantial influence on elderly individuals’ perceptions of robotic platforms. Investigations by (Bevilacqua et al., 2021; Liu et al., 2023) determined that many elderly participants referenced dystopian science fiction scenarios, including robotic rebellion, enhanced surveillance, or diminished human connection, demonstrating these cultural narratives were internalized. Even when engaging with basic assistive platforms, some participants expressed concerns about monitoring or replacement, obscuring distinctions between imagination and reality. Previous adverse technology experiences also contributed to skepticism and distrust (Fraune et al., 2022). observed that frustration with digital health applications, automated teller machines, or voice assistants fostered general reluctance to trust emerging technologies. Elderly individuals with prior negative experiences using smartphones or similar devices demonstrated a greater likelihood of perceiving robotic platforms as unreliable or emotionally detached, a distrust that often developed before any direct platform interaction.

Social and peer influences demonstrated the importance of shaping acceptance or fear (Shih et al., 2023). revealed that elderly participants were more receptive to robotic platforms when friends or caregivers demonstrated positive engagement, while negative social cues could intensify anxiety (Robinson et al., 2013). These findings suggest that robotic fear is often socially constructed, not merely an individual response. The generational digital divide further intensified apprehensive responses. Investigations (Bevilacqua et al., 2021; Destephe et al., 2015; Gomez-Hernandez, 2024; Shih et al., 2023) indicated that elderly individuals with limited digital literacy found robotic platforms more foreign and intimidating. Conversely, those comfortable with smartphones or tablets demonstrated reduced fear and greater acceptance of robotic platforms, showing that technological familiarity generally diminishes concern. While cultural and demographic elements, such as robotic appearance and interaction style, also influence apprehensive responses (Bevilacqua et al., 2021; Destephe et al., 2015; Gomez-Hernandez, 2024; Shih et al., 2023). These should be understood as contextual amplifiers rather than fundamental causes. Despite recognition of these elements, most investigations do not distinguish between immediate triggers and deeper sources of fear. A robust conceptual framework is needed to separate proximal (contextual) triggers from underlying (internalized) origins, enabling the development of emotionally intelligent and culturally sensitive robotic platforms for elderly populations.

### 5.3 Influence of fear on acceptance and utilization (RQ3)

While perceived functionality and ease of use are foundational to the Technology Acceptance Model (TAM) (Silva, 2015), this review confirms that fear is a primary emotional barrier to both the acceptance and sustained use of social robots by older adults. Unusual robot appearance, anthropomorphic traits, and privacy concerns frequently lead to discomfort, withdrawal, or outright rejection of robotic systems, even when users acknowledge their potential benefits (Patel and Rughani, 2022). Emotional authenticity and perceived surveillance are particularly important for companionship and social interaction robots, with many older adults expressing resistance due to a lack of genuine effect or concerns about being monitored (Pu et al., 2019). Moreover, digital literacy further moderates these outcomes. Older adults with lower digital confidence are more likely to avoid robot interaction in the face of intimidation or unfamiliarity (Fraune et al., 2022). In contrast, interventions featuring adaptive robot behaviors such as friendlier communication, slower movement, or personalized language have been shown to enhance trust and acceptance (Shih et al., 2023; Søraa et al., 2022) highlight the value of personalizing user interfaces and interaction parameters, especially in healthcare, to reduce anxiety and foster a sense of control. A noteworthy methodological gap remains: few studies measures baseline fear before interaction or track changes over time, leaving the trajectory of fear (whether it diminishes or intensifies with exposure) largely unknown. Although some intervention studies have measured subtle emotional shifts longitudinally (Bradwell, Hannah, 2021; Dosso et al., 2023), most focus primarily on usability rather than addressing fear as a psychological construct. Nonetheless, consistent evidence shows that familiarization sessions, peer modeling, and pre-exposure orientation can mitigate fear, even for initially reluctant users (Shih et al., 2023). These findings underscore the need to explicitly integrate affective variables, fear, trust, and emotional safety into future iterations of the Technology Acceptance Model. Transparent data usage, user control, and emotionally congruent robot behaviors are all essential for fostering acceptance. Design features such as clear privacy policies, manual overrides, and predictable, slow movements can help alleviate concerns about autonomy and surveillance, ultimately supporting both therapeutic engagement and emotional wellbeing during technology adoption.

## 6 Gaps in literature and future directions

Although research on Human–Robot Interaction (HRI) with older adults has expanded considerably, several unresolved gaps continue to limit progress in understanding fear and its implications for robot acceptance. These gaps can be grouped into three broad areas: longitudinal inquiry, cultural sensitivity, and multimodal methodologies.

Longitudinal needs: Much of the current work on fear in HRI with older adults is based on short trials or one-off encounters. These designs capture immediate impressions but cannot tell us how fear unfolds with repeated exposure. It remains unclear whether initial anxiety fades with familiarity, persists as avoidance, or develops into more complex emotional responses. Reviews of the field consistently note that longitudinal evidence is scarce and that most studies rely on brief, controlled interventions (Bradwell, 2021; Broadbent et al., 2009). To move beyond these snapshots, large-scale projects that follow participants over months or years are needed. Long-term studies in real-world care environments such as nursing homes, assisted living facilities, and private households would help clarify whether and how older adults adapt to robots in everyday life. Without this evidence, our picture of how fear develops or recedes over time remains incomplete.

Cultural dimensions: Fear of robots is not uniform across cultural contexts. While studies from East Asia often report relatively positive responses and fewer concerns about autonomy (Yam et al., 2023; Zafrani and Nimrod, 2018) work from Europe and North America highlights anxieties about privacy, surveillance, and reduced personal agency (Coco et al., 2018; Rantanen et al., 2018). Yet, systematic cross-cultural comparisons remain rare. Rather than assuming a universal emotional trajectory, future research should investigate how values, norms, and expectations shape fear-related reactions. This raises the question of whether robots should be designed with culturally specific features or whether universal design frameworks can be adapted through modular personalization. Linking Table 4 with cultural contexts would help clarify which fear categories are more salient in different regions, thereby guiding culturally responsive robot design.

Methodological and multimodal considerations: Another weakness in the current work is methodological. Heavy reliance on cross-sectional surveys and self-report scales risks underestimating implicit or nuanced forms of fear, especially in populations with cognitive decline. Few studies include older adults with moderate-to-severe dementia, despite the frequent use of robots in dementia care (Baisch et al., 2017; Dosso et al., 2023). Multimodal approaches that integrate physiological markers, such as galvanic skin response, heart rate variability, eye-tracking, behavioral observation, and interviews, would capture both overt reactions and subtle affective states. Mixed methods design combining these measures with qualitative accounts can uncover how fear is experienced, narrated, and expressed in different settings (Zafrani et al., 2023). Importantly, most existing studies have been conducted in controlled laboratory environments. Longitudinal ethnographic research in naturalistic care settings would provide richer insights into the ways fear manifests in everyday interactions.

Emerging tools: Virtual reality (VR) offers a promising avenue for advancing fear research in HRI. Controlled simulations allow researchers to vary robot appearance, behaviors, and potential malfunctions without exposing participants to physical risks. This is especially useful for investigating phenomena such as the Uncanny Valley or responses to unexpected breakdowns. VR can also support iterative prototyping before robots are physically deployed. However, its use in older populations requires caution, as VR headsets may induce discomfort or fail to replicate the complexity of real-world interaction.

Stratification and diversity: Fear in HRI is not monolithic; it varies across age brackets, cognitive status, and prior experience. Early evidence suggests that younger cohorts of older adults (65–74) often express anxiety linked to unfamiliarity, whereas those over 75 are more likely to highlight privacy or autonomy concerns (Baisch et al., 2017; Bradwell, 2021). Stratified analyses by age, cognitive condition, and cultural background are essential to develop context-aware, emotionally adaptive robots that address diverse needs.

## 7 Conclusion

This scoping review examined 49 studies published between 2014 and 2025 on older adults’ experiences of fear when interacting with robots. The findings suggest that fear is expressed in multiple ways, including worries about privacy, trust, dependence, emotional unease, and the Uncanny Valley effect. These responses were shaped by factors such as prior technology use, age, cognitive condition, and cultural context. For instance, participants with greater digital experience tended to report less fear, while studies from Western settings often emphasized privacy and surveillance concerns. Taken together, the evidence provides a broad map of how fear manifests in HRI and where future work should focus.

Limitations: The review has several limitations. Some relevant research may not have been captured, especially studies reported in non-English outlets. The included studies were highly diverse in design and outcome measures, which limited systematic comparison. Few papers involved participants with significant cognitive impairment, leaving questions about this group unanswered. Finally, as a scoping review, no formal grading of study quality was conducted, meaning that the strength of evidence cannot be ranked.

Implications and future directions: Despite these limitations, this review makes three key contributions. It consolidates evidence on the forms and triggers of fear in HRI, it highlights major gaps such as the scarcity of longitudinal and culturally comparative work, and it provides a framework for integrating multimodal methods into future studies. For designers, the results point to the value of transparent, user-informed design that avoids deceptive human-like cues. For care providers, gradual exposure and supportive introduction can help reduce initial anxiety. For policymakers, the findings underscore the need for culturally sensitive guidelines that balance innovation with the emotional wellbeing of older adults. Rather than viewing fear only as an obstacle, it should be treated as a design signal that can inform the development of robots that are transparent, trustworthy, and responsive to the needs of older adults. Confronting these fears directly is essential if robots are to be integrated into eldercare in ways that are both safe and genuinely supportive.

## Data Availability

The original contributions presented in the study are included in the article/Supplementary Material, further inquiries can be directed to the corresponding author.
