# Supplementary material for: Exploring fear in human-robot interaction: a scoping review of older adults’ experiences with social robots
Source: Front Robot AI. 2025 Oct 13;12:1626471. doi: 10.3389/frobt.2025.1626471 (PMC12554585; doi:10.3389/frobt.2025.1626471)
Supplement: Supplementary file 1 [file DataSheet2.pdf]

Supplementary Material 2: *Data Extraction Form*

Study Identification

• Study:

• Authors:

• Year of publication:

• Title:

• Journal/Source:

• Country of origin:

• Funding source:

Study Characteristics

• Study design (Quantitative/Qualitative/Mixed methods):

• Study type (Experimental/Observational/Survey/Interview/Focus group/Case study):

• Study duration (Duration of data collection period):

• Setting: (Laboratory/Care facility/Home/Multiple):

• Aims/Objectives (Primary aims of the study):

Participant Characteristics

• Sample size (Total number of participants):

• Age range (Minimum-Maximum):

• Mean age (Mean age of participants):

• Cognitive status (If reported):

• Health status (If reported):

• Prior technology experience (If reported):

• Inclusion criteria (As reported in the study):

• Exclusion criteria (As reported in the study):

Robot Characteristics

• Robot name/model (Specific name or model):

• Robot type (Humanoid/Pet-like/Service/Multiple):

• Appearance description (Physical characteristics):

• Functionality (Capabilities and functions):

- Purpose (Intended use):
- Interaction modalities (Voice/Touch/Gesture/Multiple):

Fear Assessment

- Assessment method (Questionnaire/Physiological measures/Behavioral observation/Interview):
- Validated instruments used (Names of instruments):
- Timing of assessment (Pre-interaction/During interaction/Post-interaction/Longitudinal):
- Specific measures (Details of what was measured):

Types of Fear Responses

- Privacy and data security concerns (Yes/No, with details):
- Fear of physical harm (Yes/No, with details):
- Fear of dependency and loss of autonomy (Yes/No, with details):
- Fear of dehumanized care (Yes/No, with details):
- Other fear types (If reported):
- Physiological manifestations (If measured):

Contributing Factors

- Robot design and appearance factors:
- Prior technology experience influence:
- Cultural background influence:
- Robot behavior and predictability influence:
- Perceived control influence:
- Environmental context influence:
- Other factors:

Impact on Acceptance and Utilization

- Adoption intentions (If reported):
- Usage patterns (If reported):
- Long-term acceptance (If reported):
- Mediating factors (If reported):

Mitigation Strategies

- Personalization and adaptability (If implemented):
- Gradual exposure and familiarization (If implemented):

- Transparent communication (If implemented):
- User control mechanisms (If implemented):
- Balanced design approach (If implemented):
- Other strategies (If implemented):
- Effectiveness of strategies (Results reported):

Key Findings

- Main results: (Summary of primary findings):
- Statistical significance: (If reported):
- Effect sizes: (If reported):
- Qualitative themes: (If reported):

Quality Assessment

- MMAT score (Score from quality assessment):
- Methodological strengths (Noted strengths):
- Methodological limitations (Noted limitations):

Notes

- Reviewer comments (Any additional notes):
- Follow-up needed (Any clarifications required):
