# Supplementary material for: Exploring fear in human-robot interaction: a scoping review of older adults’ experiences with social robots
Source: Front Robot AI. 2025 Oct 13;12:1626471. doi: 10.3389/frobt.2025.1626471 (PMC12554585; doi:10.3389/frobt.2025.1626471)
Supplement: Supplementary file 2 [file DataSheet4.pdf]

Supplementary Material 4: Word Cloud illustrations

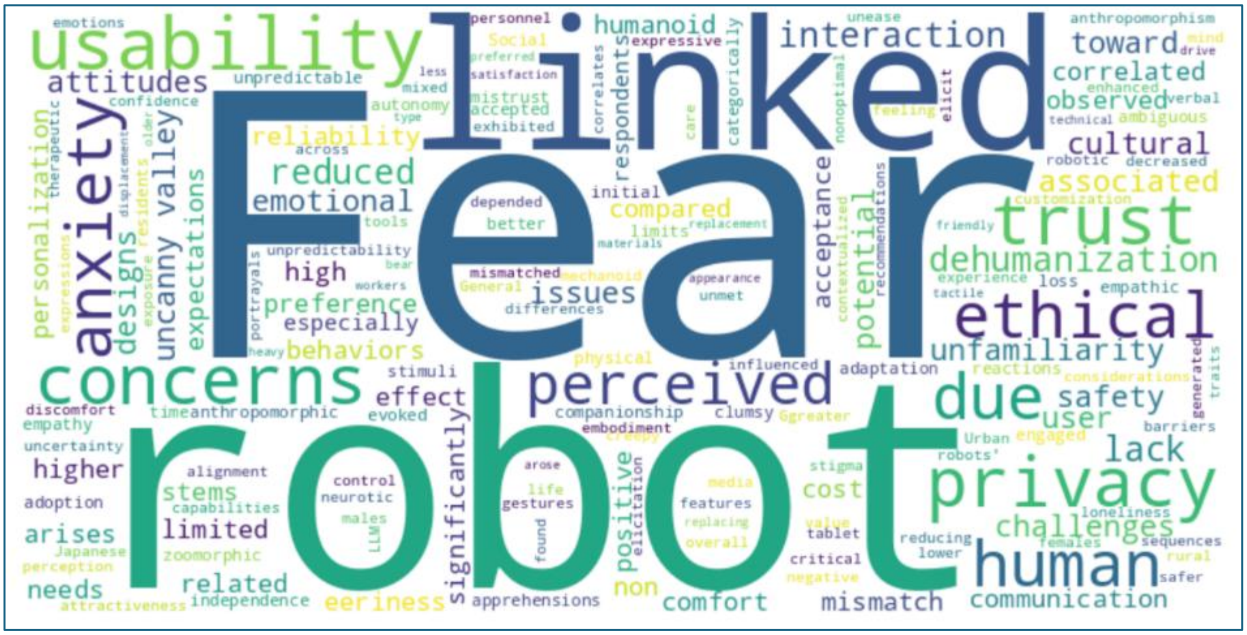

Figure 1: Word cloud highlighting dominant fear-related themes in older adults' interactions with robots, emphasizing trust, privacy, usability, and ethical concerns.

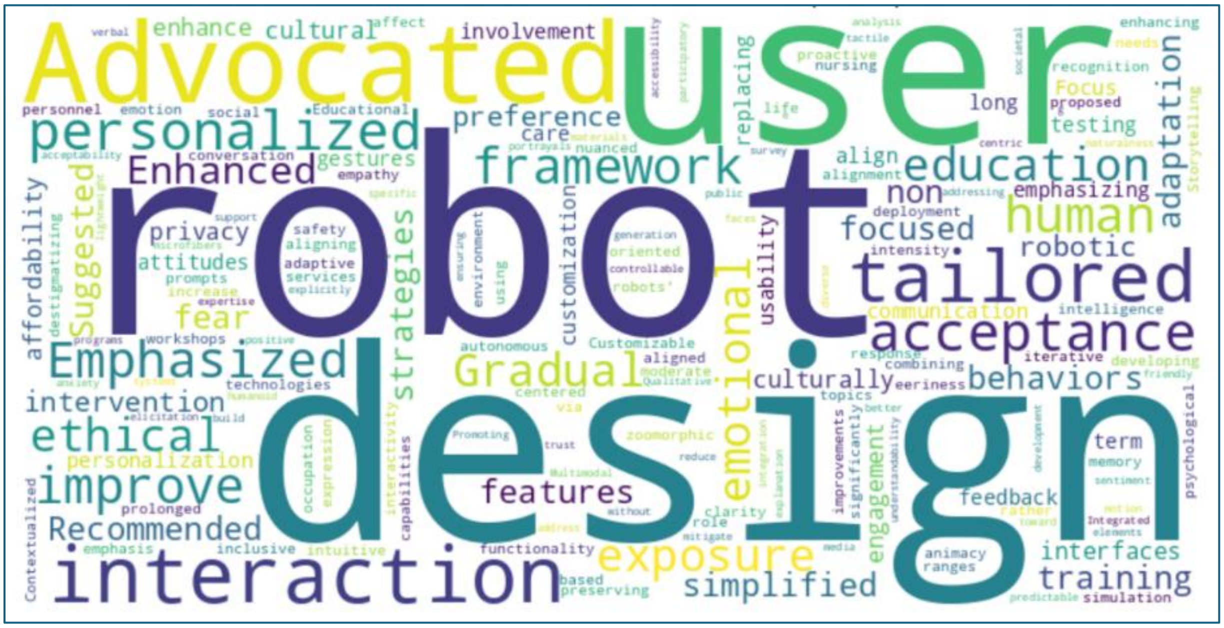

Figure 2: Word cloud highlighting dominant fear-related themes in older adults' interactions with robots, emphasizing trust, privacy, usability, and ethical concerns.
