# Supplementary material for: Exploring fear in human-robot interaction: a scoping review of older adults’ experiences with social robots
Source: Front Robot AI. 2025 Oct 13;12:1626471. doi: 10.3389/frobt.2025.1626471 (PMC12554585; doi:10.3389/frobt.2025.1626471)
Supplement: Supplementary file 3 [file DataSheet3.pdf]

Supplementary Material 3: Quality Assessment Results Using Mixed Methods Appraisal Tool (MMAT)

Summary of Quality Assessment

- High quality studies (n=11, 22.4%)
- Moderate quality studies (n=34, 69.4%)
- Low quality studies (n=4, 8.2%)

Quantitative Studies (n=26)

Study	Clear Research Question	Appropriate Sampling	Representative Sample	Appropriate Measurements	Acceptable Response Rate	Overall Rating
Chen et al., 2019	Yes	Yes	Yes	Yes	Yes	High
Tobis et al., 2022	Yes	Yes	Yes	Yes	No	Moderate
Tschöpe et al., 2017	Yes	Yes	No	Yes	Yes	Moderate
Strutz et al., 2024	Yes	Yes	Yes	Yes	Yes	High
Yuan et al., 2024	Yes	Yes	No	Yes	Yes	Moderate
Sun & Ye, 2024	Yes	Yes	Yes	Yes	Yes	High
Zafrani, 2022	Yes	Yes	Yes	Yes	Yes	High
Park et al., 2021	Yes	Yes	Yes	Yes	Yes	High
Lubold et al., 2016	Yes	Yes	Yes	Yes	Yes	High
Others	Varied	Varied	Varied	Varied	Varied	Varied

Qualitative Studies (n=11)

Study	Appropriate Approach	Adequate Data Collection	Findings Derived from Data	Data Sufficiently Substantiates Findings	Researchers' Influence Addressed	Overall Rating
Zafrani et al., 2022	Yes	Yes	Yes	Yes	No	Moderate
Vozna & Costantini, 2025	Yes	Yes	Yes	Yes	Yes	High
Sharkey & Sharkey, 2012	Yes	No	Yes	No	No	Low
Carros et al., 2020	Yes	Yes	Yes	Yes	Yes	High
Rigaud et al., 2024	Yes	Yes	Yes	Yes	Yes	High
Søraa et al., 2022	Yes	Yes	Yes	Yes	Yes	High
Deutsch et al., 2019	Yes	Yes	Yes	Yes	No	Moderate
Moyle et al., 2019	Yes	Yes	Yes	Yes	No	Moderate
Vandemeulebroucke et al., 2019	Yes	Yes	Yes	Yes	No	Moderate
Leung et al., 2023	Yes	Yes	Yes	Yes	Yes	High
Yam et al., 2023	Yes	Yes	Yes	Yes	No	Moderate

Mixed Methods Studies (n=6)

Study	Adequate Rationale	Integration of Components	Integration Adequately Interpreted	Divergences Addressed	Quality of Components Maintained	Overall Rating
Baisch et al., 2017	Yes	Yes	Yes	Yes	Yes	High
Robinson et al., 2014	Yes	Yes	No	No	Yes	Moderate
Zhao et al., 2023	Yes	Yes	Yes	No	Yes	Moderate
Appel et al., 2019	Yes	Yes	Yes	Yes	Yes	High
Antona et al., 2019	Yes	Yes	Yes	No	Yes	Moderate
Dosso et al., 2023	Yes	Yes	Yes	Yes	Yes	High

Other Designs or Theoretical/Ethical Analyses (n=6)

Study	Clear Objective	Methodological Framework	Evidence Integration	Relevance to Fear Constructs	Overall Rating
Miklósi et al., 2017	Yes	Conceptual Review	Yes	High	High
Pino et al., 2015	Yes	Ethical-Theoretical	Yes	High	Moderate
Yam et al., 2023	Yes	Comparative-Theoretical	Partial	Moderate	Moderate
Vozna & Costantini, 2025	Yes	Narrative with Case Study	Yes	High	High
Yamaguchi, 2025	Yes	Experimental (IAT/Affective Priming)	Yes	High	High
Wu et al., 2014	Yes	Mixed Conceptual-Qualitative	Partial	Moderate	Moderate

Common Methodological Limitations

1. Small Sample Sizes
   - 18 studies (36.7%) had fewer than 30 participants
   - Limited statistical power in quantitative analyses
   - Restricted generalizability of findings
2. Convenience Sampling
   - 26 studies (53.1%) used convenience sampling
   - Potential selection bias toward technology-accepting participants
   - Limited representation of diverse older adult populations
3. Lack of Validated Fear Assessment Tools
   - 22 studies (44.9%) used non-validated or ad hoc measures
   - Inconsistent operationalization of fear constructs
   - Challenges in comparing results across studies
4. Limited Consideration of Confounding Factors
   - 19 studies (38.8%) did not adequately control for confounding variables
   - Insufficient accounting for prior technology experience
   - Limited consideration of cognitive status and health conditions
5. Short Interaction Periods
   - 31 studies (63.3%) involved single or brief interactions with robots
   - May not capture the evolution of fear responses over time
   - Limited ecological validity compared to real-world implementation

Note: This quality assessment used the Mixed Methods Appraisal Tool (MMAT) version 2018 (Hong et al., 2018). The MMAT appraises diverse methodological designs and supports consistency in evaluating heterogeneous research evidence.
