# Supplementary material for: Exploring fear in human-robot interaction: a scoping review of older adults’ experiences with social robots
Source: Front Robot AI. 2025 Oct 13;12:1626471. doi: 10.3389/frobt.2025.1626471 (PMC12554585; doi:10.3389/frobt.2025.1626471)
Supplement: Supplementary file 4 [file DataSheet1.pdf]

Supplementary Material 1: PRISMA-ScR Checklist Item

Section	Item	PRISMA-ScR Checklist Item	Location in Manuscript
TITLE			
Title	0	Identify the report as a scoping review.	Title page
ABSTRACT			
Structured summary	1	Provide a structured summary that includes (as applicable) background, objectives, eligibility criteria, sources of evidence, charting methods, results, and conclusions related to the review questions and objectives.	Abstract section
INTRODUCTION			
Rationale	2	Describe the rationale for the review in the context of what is already known. Explain why the review questions/objectives lend themselves to a scoping review approach.	Section 2.1-2.4
Objectives	3	Provide an explicit statement of the questions and objectives addressed regarding their key elements (e.g., population or participants, concepts, and context) or other relevant key elements used to conceptualize the review questions and/or objectives.	Section 3
METHODS			
Protocol and registration	4	Indicate whether a review protocol exists; state if and where it can be accessed (e.g., a Web address); and provide registration information, including the registration number if available.	Section 4.1
Eligibility criteria	5	Specify characteristics of the sources of evidence used as eligibility criteria (e.g., years considered, language, and publication status) and provide a rationale.	Section 4.2
Information sources	6	Describe all information sources in the search (e.g., databases with dates of coverage and contact with authors to identify additional sources) and the date the most recent search was executed.	Section 4.3
Search	7	Present the complete electronic search strategy for at least one database, including any limits used, so it could be repeated.	Section 4.4, Appendix A
Selection of sources of evidence	8	State the process for selecting sources of evidence (i.e., screening and eligibility) included in the scoping review.	Section 4.5
Data charting process	9	Describe the methods of charting data from the included sources of evidence (e.g., calibrated forms or forms that the team has tested before their use, and whether data charting was done independently	Section 4.6

Section	Item	PRISMA-ScR Checklist Item	Location in Manuscript
		or in duplicate) and any processes for obtaining and confirming data from investigators.	
Data items	10	List and define all variables for which data were sought and any assumptions and simplifications made.	Section 4.7
Critical appraisal of individual sources of evidence	11	If done, provide a rationale for conducting a critical appraisal of included sources of evidence; describe the methods used and how this information was used in any data synthesis (if appropriate).	Section 4.7
Synthesis of results	12	Describe the methods of handling and summarizing the data that were charted.	Section 4.8
RESULTS			
Selection of sources of evidence	13	Give numbers of sources of evidence screened, assessed for eligibility, and included in the review, with reasons for exclusions at each stage, ideally using a flow diagram.	Section 5.1, Figure 1
Characteristics of sources of evidence	14	For each source of evidence, present characteristics for which data were charted and provide the citations.	Section 5.2, Table 1
Critical appraisal of sources of evidence	15	If done, present data on critical appraisal of included sources of evidence (see item 12).	Section 4.3
Results of individual sources of evidence	16	For each included source of evidence, present the relevant data that was charted that relates to the review questions and objectives.	Section 4.4
Synthesis of results	17	Summarize and/or present the charting results related to the review questions and objectives.	Section 4.5, Figure 3, Table 2
DISCUSSION			
Summary of evidence	18	Summarize the main results (including an overview of concepts, themes, and types of evidence available), link to the review questions and objectives, and consider the relevance to key groups.	Section 5.1-5.4
Limitations	19	Discuss the limitations of the scoping review process.	Section 5.5-5.6
Conclusions	20	Provide a general interpretation of the results concerning the review questions, objectives, and potential implications and/or next steps.	Section 6
FUNDING			
Funding	21	Describe funding sources for the included sources of evidence and the scoping review. Describe the role of the funders of the scoping review.	Not applicable
