# Supplementary material for: Exploring fear in human-robot interaction: a scoping review of older adults’ experiences with social robots
Source: Front Robot AI. 2025 Oct 13;12:1626471. doi: 10.3389/frobt.2025.1626471 (PMC12554585; doi:10.3389/frobt.2025.1626471)
Supplement: Supplementary file 5 [file DataSheet5.pdf]

Appendices

Appendix A: Complete Search Strategy

PubMed:

(("older adult*"[Title/Abstract] OR "elderly"[Title/Abstract] OR "senior*"[Title/Abstract] OR "aged"[MeSH Terms] OR "geriatric*"[Title/Abstract] OR "aging"[Title/Abstract] OR "ageing"[Title/Abstract] OR "older people"[Title/Abstract] OR "older individual*"[Title/Abstract] OR "senior citizen*"[Title/Abstract] OR "older persons"[Title/Abstract]) AND ("robot*"[Title/Abstract] OR "humanoid*"[Title/Abstract] OR "social robot*"[Title/Abstract] OR "assistive robot*"[Title/Abstract] OR "companion robot*"[Title/Abstract] OR "care robot*"[Title/Abstract] OR "eldercare robot*"[Title/Abstract] OR "elder care robot*"[Title/Abstract] OR "geriatric robot*"[Title/Abstract] OR "robotic assistant*"[Title/Abstract] OR "robotic companion*"[Title/Abstract] OR "robotic care*"[Title/Abstract] OR "service robot*"[Title/Abstract] OR "socially assistive robot*"[Title/Abstract] OR "SAR"[Title/Abstract] OR "human-robot interaction"[Title/Abstract] OR "HRI"[Title/Abstract])

AND ("fear*"[Title/Abstract] OR "anxiety"[Title/Abstract] OR "anxious"[Title/Abstract] OR "discomfort"[Title/Abstract] OR "negative emotion*"[Title/Abstract] OR "negative response*"[Title/Abstract] OR "negative reaction*"[Title/Abstract] OR "acceptance"[Title/Abstract] OR "acceptability"[Title/Abstract] OR "rejection"[Title/Abstract] OR "attitude*"[Title/Abstract] OR "perception*"[Title/Abstract] OR "uncanny valley"[Title/Abstract] OR "technophobia"[Title/Abstract] OR "robophobia"[Title/Abstract] OR "technology anxiety"[Title/Abstract] OR "technological anxiety"[Title/Abstract] OR "robot fear"[Title/Abstract] OR "robot-induced stress"[Title/Abstract] OR "technology-related distress"[Title/Abstract] OR "distrust in robots"[Title/Abstract] OR "hesitation toward robots"[Title/Abstract] OR "psychological distress"[Title/Abstract] OR "apprehension"[Title/Abstract] OR "concern*"[Title/Abstract] OR "worr*"[Title/Abstract]))

IEEE Xplore:

("older adult" OR "elderly" OR "senior" OR "aged" OR "geriatric" OR "aging" OR "ageing") AND

("robot" OR "humanoid" OR "social robot" OR "assistive robot" OR "companion robot" OR "service robot" OR "socially assistive robot" OR "human-robot interaction" OR "HRI") AND ("fear" OR "anxiety" OR "anxious" OR "discomfort" OR "negative emotion" OR "acceptance" OR "rejection" OR "attitude" OR "perception" OR "uncanny valley" OR "technophobia" OR "robophobia")

ACM Digital Library:

(("older adult*" OR "elderly" OR "senior*" OR "aged" OR "geriatric*" OR "aging" OR "ageing")

AND ("robot*" OR "humanoid*" OR "social robot*" OR "assistive robot*" OR "companion robot*" OR "service robot*" OR "socially assistive robot*" OR "SAR" OR "human-robot interaction" OR "HRI")

AND ("fear*" OR "anxiety" OR "anxious" OR "discomfort" OR "negative emotion*" OR "negative response*" OR "negative reaction*" OR "acceptance" OR "acceptability" OR "rejection" OR "attitude*" OR "perception*" OR "uncanny valley" OR "technophobia" OR "robophobia"))

PsycINFO:

((DE "Aging" OR DE "Elder Care" OR DE "Geriatrics" OR TI ("older adult" OR "older adults" OR "elderly" OR "senior" OR "seniors" OR "aged" OR "geriatric" OR "aging" OR “ageing” OR "older people") AND (DE "Robotics" OR DE "Assistive Technology" OR TI ("robot*" OR "humanoid" OR "social robot" OR "assistive robot" OR "companion robot" OR "service robot" OR "human-robot interaction" OR "HRI")) AND (DE "Fear" OR DE "Anxiety" OR DE "Technology Acceptance" OR DE "Technophobia" OR TI ("fear" OR "anxiety" OR "acceptance" OR "attitude" OR "perception" OR "uncanny valley" OR "robophobia" OR "technophobia"))

Web of Science:

(“older adult*" OR "elderly" OR "senior*" OR "aged" OR "aging population" OR "ageing population" OR "geriatric*" OR "older people" OR "older individuals" OR "older persons" OR "senior citizen*"

)AND ("robot*" OR "humanoid robot*" OR "social robot*" OR "assistive robot*" OR "companion robot*" OR "care robot*" OR "eldercare robot*" OR "elder care robot*" OR "geriatric robot*" OR "robotic assistant*" OR "robotic companion*" OR "robotic care*" OR "service robot*" OR "socially assistive robot*" OR "SAR" OR "human-robot interaction" OR "HRI") AND ("fear*" OR "anxiety" OR "anxious" OR "discomfort" OR "negative emotion*" OR "negative response*" OR "negative reaction*" OR "psychological impact" OR "trust" OR "emotional response*" OR "technology acceptance" OR "acceptance" OR "acceptability" OR "rejection" OR "attitude*" OR "perception*" OR "uncanny valley" OR "technophobia" OR "robophobia" OR "technology anxiety" OR "technological anxiety" OR "robot fear" OR "robot-induced stress" OR "technology-related distress" OR "distrust in robots" OR "hesitation toward robots" OR "psychological distress" OR "apprehension" OR "concern*" OR "worr*")

Scopus (Elsevier)

(TITLE-ABS-KEY("older adult*" OR "elderly" OR "senior*" OR "aged" OR "geriatric*" OR "aging" OR "ageing") AND ("robot*" OR "humanoid*" OR "social robot*" OR "assistive robot*" OR "companion robot*" OR "service robot*" OR "socially assistive robot*" OR "SAR" OR "human-robot interaction" OR "HRI") AND ("fear*" OR "anxiety" OR "anxious" OR "discomfort" OR "negative emotion*" OR "negative response*" OR "negative reaction*" OR "acceptance" OR "acceptability" OR "rejection" OR "attitude*" OR "perception*" OR "uncanny valley" OR "technophobia" OR "robophobia"))

Appendix B

Table 1: Table of studies

Author/s	Year	Country	Study Design	Sample Size	Age Range	Robot Type	Fear Interventions	Fear Assessment Tools	Publisher	Study Type	Key Findings Related to Fear of Robots
(Antona et al., 2019)	2019	Greece	Mixed method	32	28-94	RAMCIP	Developed affective output via 2D facial expressions and prosody; tested emotion recognition and speech perception.	Emotion recognition tests (Ekman 60 Faces Test), matching speech prosody to facial expressions, qualitative user feedback, pilot testing in a home environment	Proceedings of PErvasive Technologies Related to Assistive Environments (PETRA 2019)	Empirical	Users effectively recognized emotional valence; interaction slightly improved emotion recognition, with expressiveness valued.
(Appel et al., 2019)	2019	Germany	Mixed method	406	50-65	Ellix	Robots with a mind (experience) elicit higher eeriness than robots as tools.	Contextualized in a nursing environment	Elsevier (Computers in Human Behavior	Empirical	Robots with a mind (experience) elicit higher eeriness than robots as tools.
(Backonja et al., 2018)	2018	USA	Mixed method	499	18–98	General robots	NARS and social impact/comfort questions; exposure varied based on tech familiarity	Negative Attitudes Toward Robots Scale (NARS), social impact scale, comfort scale, open-ended comments	Journal of Nursing Scholarship	Empirical	Fear stemmed from unfamiliarity and media influence; robots were preferred as support tools. Gender, tech access, and confidence shaped fear more than age
(Baisch et al., 2017)	2017	Germany	Mixed method	29	65–81	Paro & Giraff	Gradual exposure, personalization	Heerink's anxiety and ease of use scales; intention-to-use items; psychosocial metrics (GDS, loneliness, satisfaction, support	International Journal of Social Robotics (Springer)	Empirical	Fear and acceptance depended on user–tech fit; Giraff faced low acceptance with poor psychosocial function, while Paro evoked stigma from identity mismatch.
(Berns & Ashok, 2024)	2024	Germany	Mixed Method	116	18-85	EMAH, ROMAN, ROBIN	Interactive experience	Likert Scales, Godspeed Questionnaire, Emotion Recognition, Open-ended responses, Descriptive Statistics	Actuators (MDPI)	Empirical	EMAH's expressiveness boosted trust; rigid, mismatched robots like ROMAN triggered uncanny responses. Emotion-rich design helped reduce fear.
(Carros et al., 2020)	2020	Germany	Qualitative	29	65-89	Temi	Long-term exposure, co-designed, personalized interaction	Observations, video recordings, semi-structured interviews, thematic analysis	Springer	Empirical	Initial fear decreased with familiarity; unpredictability triggered fear, and caregiver support built trust. Robots were seen as companions, not replacements.

(Cavallo et al., 2018)	2018	Italy	Mixed method	45	65–86	Robot-Era (3 robots: DORO, CORO, ORO)	Familiar environment	Appearance Questionnaire, Ad hoc Acceptability Questionnaire, System Usability Scale (SUS)	Journal of Medical Internet Research	Empirical	Anxiety was low; trust increased through guided interaction, clear robot functions, and approachable design. Initial fears about privacy or complexity faded with familiarity and training.
(Coco et al., 2018)	2018	Finland	Cross-sectional comparative survey	286	43-65	Zora, Paro, Double, RIBA	Education and early staff involvement in design are suggested to reduce fear.	Likert-scale questionnaire (15 items), Mann–Whitney U test	Journal of Nursing Scholarship	Empirical	Japanese staff were more accepting; Finnish staff feared dehumanization and job loss. Both supported robots for safety, not personal care. Culture shaped attitudes.
(Conde et al., 2024)	2024	Germany	Qualitative	30	60–74	Telepresence Robot (CO-HUMANICS prototype, storyboards used)	Scenario exposure, discussions on autonomy, privacy, and control	Thematic analysis from interviews (MAXQDA)	International Journal of Social Robotics	Empirical	Attitudes ranged from enthusiastic to reluctant. Fears focused on control, privacy, cost, and complexity. Benefits included companionship and aging-in-place. Trust, autonomy, and usability drove acceptance.
(Deutsch et al., 2019)	2019	Austria	Qualitative	30	67-90	PR2, Nao, ElliQ, Cozmo, Paro, Google Home	Post-exposure to robot videos	Thematic analysis	Computers in Human Behavior	Empirical	Fear stemmed from loss of control and inauthenticity; small, functional robots respecting autonomy were bette(Dosso et al., 2023)(Dosso et al., 2023)
(Dosso et al., 2023)	2023	Canada	Qualitative	44.0	50-80	MiRo & T-Top	Video demos, group discussion, emotional alignment design, public use concerns	MDRAS, PIADS, Zoom polls, thematic analysis	Frontiers in Psychiatry	Empirical	Fear arose from stigma, control loss, and emotional mismatch; trust improved with adaptive, context-aware robots. Pet-like designs were preferred in private settings was minimized.
(Fraune et al., 2020)	2020	USA	Mixed method	701	21-65	Sociable Trash Box (STB) – minimally social robots	Evaluated behavior effects through social/functional HRI interactions	Anthropomorphism scales & Godspeed, attitude & emotion scales	Computers in Human Behavior (Elsevier)	Empirical	Social robot-human behavior boosted trust and interaction. Group entitativity raised anthropomorphism. Functional roles reduced warmth. Robot-robot sociality felt humanlike but less comforting. Real-world effects surpassed video.
(Gasteiger et al., 2025)	2022	New Zealand	Mixed method	119	54-101	Bomy & Silbot	Co-design, long-term use, personalization, cultural alignment	Thematic interviews, acceptability/usability questionnaires, observational logs	ACM Transactions on Human-Robot Interaction	Empirical	Robots were accepted for reminders and games; stigma and unfamiliarity caused hesitation: personalization and long-term use-built trust. Physical robots offered preferred companionship.
(Görer et al., 2017)	2017	Turkey	Mixed method	12	70–88	Aldebaran NAO	Personalized feedback, repeated exposure, verbal guidance,	Motion tracking, error analysis, video coding (gaze, expression, interaction),	Autonomous Robots (Springer)	Empirical	Initial hesitation faded with exposure. Feedback and gamification boosted engagement. Concerns included unclear speech and dependence. Cultural views limited companionship. Personalized cues improved trust.

							gamified scoring, facial recognition	Likert-based surveys			
(Harrison, 2015)	2015	Netherlands	Mixed method	3	65-78	Alice & Robokind & (SELEMCA project prototype)	Cartoon-like humanoid design to avoid uncanny valley; gradual home exposure; designed for emotional support and loneliness mitigation.	Anxiety scale, behavioral observations	The Lancet	Empirical	Initial resistance faded as emotional engagement grew. Most participants accepted the robot for companionship and reminders, though some skepticism remained. Familiarity and emotional design helped reduce fear.
(Huang et al., 2024)	2024	USA	Mixed method	22	18-74	GR-1 humanoid robot	Technology familiarization	7-point Likert scales & Qualitative Interviews	HRI/Robotics	Empirical	Emotion gestures outperformed both AI-only and human demos. Trust rose with more precise, realistic motion. Naturalness was shaped by pose, timing, and symmetry. Higher empathy improved ratings: age slightly reduced them. Clear, culturally aligned gestures help reduce fear.
(I. Giorgi et al., 2022)	2022	UK	Mixed method	17	40–87	NAO	Manipulated attitude	Trust scale (6 items), intention-to-use Likert scale, qualitative interviews, video-coded reactions	IEEE Access	Empirical	Trust dropped with errors despite warmth; warmth helped only when error-free. Trust strongly predicted willingness to use. Reactions to robot touch were mixed—some positive, others hesitant.
(Jung et al., 2017)	2017	Netherlands	Mixed method	9	24–67	Paro	Fears were explored via interviews and touch gesture ratings. Key concerns included overstimulation, deception, and infantilization.	Likert-scale gesture ratings, qualitative thematic analysis	Frontiers in ICT	Empirical	Paro promoted calm through touch, but auditory and design issues raised concerns. Advanced tactile feedback was seen as helpful yet potentially overstimulating.
(Koceski & Koceska, 2016)	2016	Macedonia	Mixed method	35	65-78	Assistive Telepresence Robot	Telepresence functionalities, navigation, video conferencing, manipulator tasks, and reminder systems	Modified Technology Acceptance Model (TAM) questionnaire, interviews	Journal of Medical Systems	Empirical	Telepresence robot functionalities were generally perceived as valuable and easy to use, though caregivers rated vital signs and reminder functionalities higher than elderly participants. The elderly found video conferencing particularly valuable for reducing loneliness.
(Leung et al., 2022)	2022	Hong Kong	Qualitative	4	65-86	Ka Ka	Long-term in-home use and personalization	Semi-structured interviews, usage logs, and thematic analysis	Healthcare (MDPI)	Empirical	Ka Ka eased loneliness, enriched routines, and supported family bonds. I accepted as a companion, but tech anxiety and interface issues triggered fear. Personalization and a soft voice enhanced comfort.
(Lubold et al., 2016)	2016	USA	Quantitative	43	18-30	LEGO Mindstorms + iPod robot (Quinn)	control, social dialogue only, and voice-adaptive + social dialogue.	Rapport Scale & Social Presence Inventory	IEEE Xplore	Empirical	Voice-adaptive, expressive interaction boosted trust, engagement, and social presence, especially among females, indirectly reducing fear.
(Miklósi et al., 2017)	2017	Hungary	Conceptual Framework	N/A	N/A	Androids &	Designing robots to avoid the	Theoretical critique of	Frontiers in Psychology	Theoretical	Human likeness triggers rejection via the Uncanny Valley. Ethorobotics suggests a dog-

						Ethorobots	Uncanny Valley by mimicking animal-like social competence, not human likeness	existing models (Uncanny Valley, social competence, niche theory			like, function-driven design to reduce fear, focusing on behavior over appearance to build trust.
(Moyle et al., 2019)	2019	Australia	Qualitative	138	60–94	PARO	10-week intervention, repeated exposure, unassisted individual sessions, responsive behavior coding	Video-coded emotion observation, RUDAS, CMAI-SF, qualitative reflection	Aging & Mental Health	Empirical	Reactions to PARO varied by mood, cognition, and preference. Some bonded; others showed distress or resistance: overstimulation and forced use reduced benefit. Person-centered, flexible use was essential. Ethical concerns arose with attachment.
(Nault et al., 2024)	2024	UK	Mixed method	20	67-83	NAO & RAI	Participatory Design, sensory feedback (visual, auditory, haptic)	NASA Task Load Index (TLX), Emotion wheel, Intrinsic Motivation Inventory (IMI), Godspeed Questionnaire	ACM Transactions on Human-Robot Interaction	Experimental and Participatory design	Key barriers identified included technology usability, learning curve, physical, and cognitive challenges. Autonomy, personalization, and familiarization strategies significantly reduced fear and increased engagement.
(Olatunji et al., 2025)	2025	USA	Mixed method	12	60-97	Stretch RE2	Gradual exposure protocol, participatory design, video-based familiarization, user-controlled interaction	SUS, NASA-TLX, MoCA, Robot Trust Questionnaire, Think-Aloud, Interviews	Frontiers in Robotics and AI	Empirical	Initial uncertainty gave way to trust and comfort through gradual exposure and autonomy features. Fear stemmed from unfamiliarity and control issues. Participants favored assistive over dominant roles and voiced preferences via interaction.
(Ostrowski et al., 2019) et al.	2019	USA	Mixed method	19	67–99	Jibo	Personalization, autonomy controls, and communal exposure. Concerns focused on privacy, autonomy, and emotional dependence.	Anxiety scale, movement perception questionnaire	ACM Transactions on Human-Robot Interaction	Empirical	Initial concerns about privacy, autonomy, and social change eased over time as participants gained confidence. Social connectedness grew, and participatory design helped mitigate fear, though some skepticism remained.
(Ostrowski et al., 2024)	2024	USA	Mixed method	28	70-94	Jibo	Long-term exposure, co-design, and card sorting	Adapted ethnographic decision tree modeling and interviews,	Frontiers in Robotics and AI	Empirical	Fear focused on privacy, autonomy loss, and unnatural interactions. While admin tasks were accepted, emotional or tracking features were rejected. Co-design clarified preferences, highlighting trust, ethics, and naturalness as key to reducing anxiety.
(Park et al., 2021)	2021	South Korea	Quantitative	135	60-82	Sil-Bot	Cognitive training included engaging, emotionally expressive interactions through Sil-Bot	MMSE-DS, SMCQ, CERAD-K, GDSSF-K	International Journal of Environmental Research and Public Health	Empirical	Robot-assisted training improved cognition and reduced depression. While fear was not directly measured, enjoyment and emotional engagement suggested reduced resistance. Emotionally expressive robots supported MCI care, especially during isolation.

							with visual, verbal, and tactile stimulation.				
(Pino et al., 2015)	2015	France	Mixed method	25	65-86	RobuLAB 10 and multiple SAR examples (e.g., Kompaï, Paro, Nexi)	Exposure to robot demos and scenarios, group discussion, and co-reflection	Almere-model-based survey, open-ended focus group content analysis	Frontiers in Aging Neuroscience	Empirical	SARs were favored for future use. Healthy adults had more concerns; MCI and caregivers were more open. Personalization and simple, non-humanoid designs supported acceptance.
(Rantanen et al., 2018)	2018	Finland	Qualitative	200	43-65	Zora, Paro	Questions perceived dehumanization, job loss, loneliness, and technological comfort. Training and workplace culture	Likert-scale survey, TPB-based items (e.g., perceived control, subjective norms), NARS adaptation	Journal of Clinical Nursing	Empirical	Feared robots may dehumanize care, increase loneliness, or threaten jobs.
(Rigaud et al., 2024)	2024	France	Qualitative	86	65-90	Pepper & Paro & Nao	Training, guidelines, and ethical frameworks.	EUnetHTA Core Model® v3.0 coding framework	Journal of Rehabilitation and Assistive Technologies Engineering	Empirical	Professionals viewed SARs positively but feared privacy loss, dehumanization, and role confusion. Trust grew with exposure. Gradual, transparent introduction and co-design were key to easing fear and supporting ethical use in eldercare.
(Søraa et al., 2022)	2022	Norway	Qualitative	22	40-80	Pepper	Participatory design, interviews, repeated exposure	Thematic analysis (NVivo), inductive coding	International Journal of Social Robotics	Empirical	Initial skepticism stemmed from tech fatigue and fiction. Trust grew with involvement. Users feared losing contact and autonomy, preferring practical over emotional roles. Early user input is vital.
(Spatola et al., 2021)	2021	Germany	Mixed method	1919	20–31	Nao, Meccanoid, Nadine, Hospi	Exposure to real and virtual robots, social vs. passive robot interaction	HRIES (Human–Robot Interaction Evaluation Scale) & comparison with NARS	International Journal of Social Robotics	Empirical	Real interaction decreased perceived disturbance (fear/unease). Introduced HRIES with Disturbance as a key dimension. Fear is tied to behavior, appearance, and sociability.
(Stafford et al., 2014)	2014	New Zealand	Mixed method	20	55-71	Peoplebot	Manipulation of the robot's virtual face (humanlike, machinelike, no face), gender (male, female), and conversational interaction	Robot Attitudes Scale (RAS), robot drawings, blood pressure (BP), heart rate, robot evaluations	International Journal of Social Robotics	Empirical	Pre-existing positive attitudes toward robots strongly predicted positive evaluations after interaction. Larger robot drawings correlated with increased anxiety as indicated by elevated systolic BP. Men tended to evaluate robots more positively than women.
(Strutz et al., 2024)	2024	Germany	Mixed method	21	65-92	Pepper	The design emphasized robot-led greetings, gestures, blinking eyes, and user	Qualitative content analysis, pre/post surveys	Zeitschrift für Gerontologie und Geriatrie	Empirical	Nonverbal cues were unexpectedly pleasant and trust-building; gestures and eye contact enhanced familiarity. Verbal interaction felt limited. Participants saw the robot as helpful for others, distancing themselves despite recognizing its potential to reduce loneliness.

							feedback options.				
(Sun & Ye, 2024)	2024	China	Quantitative	602	55-98	Technology and robots in General	The study categorized technophobia subgroups based on techno-anxiety, techno-paranoia, and privacy concerns.	Chinese version of the Technophobia Scale (3 dimensions: techno-anxiety, techno-paranoia, privacy concerns); Subjective Age Scale (4 dimensions: feel-age, look-age, do-age, interests-age)	Aging & Mental Health (Taylor & Francis	Empirical	Four technophobia profiles identified: low, medium, high, and high-privacy-concerns. Privacy concerns played a significant role in higher technophobia. Lower subjective age was associated with lower technophobia. Older age and less frequent technology use correlated with higher technophobia.
(Takayanagi et al., 2014)	2014	Japan	Experimental	30	75-95	PARO	Repeated exposure to PARO with a plush toy (Lion), naturalistic interactions	Behavioral coding & Hasegawa Dementia Scale	Frontiers in Aging Neuroscience	Empirical	PARO elicited more verbal and emotional responses than a non-reactive toy, with minimal fear. It facilitated conversation and reduced loneliness, especially among mildly/moderately demented adults.
(Thunberg et al., 2022)	2022	Sweden	Experimental	19	65+	Furhat (humanoid robot with projected facial expressions)	Engagement is encouraged through polite, human-like dialogue.	NARS (Negative Attitudes Toward Robots Scale), GODSPEED questionnaire	ACM	Empirical	Older adults viewed Furhat as low in human likeness and animacy, with NARS revealing negative attitudes, especially toward emotional interactions.
(Tobis et al., 2022)	2022	Poland	Mixed method	113	65-94	TIAGo	Emphasis on real-world interaction to reduce uncertainty	Users' Needs, Requirements and Abilities Questionnaire (UNRAQ)	Sensors	Empirical	Identified challenges and opportunities; fear of dependency was prominent; personalization reduced fears.
(Torta et al., 2014)	2014	Austria	Mixed method	8	70–95	NAO	Iterative exposure: varied assistive interaction scenarios over time	Almere Model constructs: ANX, TRUST, PS, SP, PEOU	Journal of Intelligent & Robotic Systems	Original Research Article	Participants showed low anxiety levels due to the robot's small, non-intimidating form. Trust was moderately high, especially when advice was reasonable. More prolonged exposure suggested potential emotional bonding, but enjoyment decreased over time. Fear was mitigated by familiarity and positive initial experiences.
(Vandemeulebroucke et al., 2019)	2019	Belgium	Qualitative	59	70-90	Alice	Ethical framework	Focus groups, thematic analysis	Journals of Gerontology: Social Sciences	Empirical	Older adults viewed SARs as helpful but ethically complex—feared loss of control, surveillance, and emotional inauthenticity.
(Vozna & Costantini, 2025)	2025	Italy	Narrative Review with Case Analysis	24	70-92	AI-driven social robots	Ethical design emphasizing co-design, transparency, and anthropomorphism control helps reduce fear.	Qualitative synthesis	Intelligenza Artificiale	Theoretical perspective	Identify key fear drivers: privacy invasion, autonomy loss, emotional overattachment, and opacity. Recommends ethical frameworks and co-design to build trust. Acceptance improves with transparency and user involvement.
(Wu et al., 2014)	2014	France	Mixed methods	11	76–85	Kompaï	4-session exposure, guided use, usability	Robot-acceptance questionnaire (adapted	Clinical Interventions in Aging	Empirical	The robot was seen as easy and enjoyable, but their intention to use them remained low. Fears included stigma, dependence, and tech

							tasks, user feedback, social influence discussion	UTAUT), performance time/error tracking, thematic interviews			unfamiliarity. Interaction reduced mystery but not fear. Social influence may support acceptance.
(Yam et al., 2023)	2023	USA	Comparative	80	65-88	Nao& Chatbot robots & Robot priests	Eastern co-existence views and animism reduce anxiety, while Western threat narratives heighten fear. Early exposure helps mitigate fear.	Cross-cultural acceptance questionnaire	ACM/IEEE International Conference on HRI	Theoretical perspective	Cultural differences in robot acceptance: US participants showed more significant privacy concerns than Japanese participants
(Yamaguchi, 2025)	2025	UK	Qualitative	322	65-85	Robot face images	Implicit affective responses and facial likability to uncover the uncanny valley	Affective Priming, Single-Category IAT, Likability Ratings	International Journal of Human-Computer Studies	Empirical	Demonstrated uncanny valley effect via explicit (likability) and implicit affective measures (priming & IAT). Found item-level discomfort peaks with semi-humanlike faces. Priming captured fear more robustly than IAT. First, these measures can detect fear of robots to individual robot faces, not just categories. Validates affective priming as a reliable behavioral indicator of fear.
(Yuan et al., 2024)	2024	China	Experimental	60	20-60	Nao	Designed three levels of human-robot relationship (Familiar, Acquaintance, Stranger) with different dialogue styles	7-point Likert scale measuring Trust, Competence, and Acceptance	SAGE Open	Empirical	Personalized interactions reduced fear of robots; trust-building features enhanced acceptance.
(Zafrani, 2022)	2022	USA	Quantitative	120	65-90	Gymmy	Building trust through transparency, addressing technophobia, and age-related digital anxiety	NARS Technology acceptance questionnaire	Journal of Applied Gerontology	Empirical	Factors influencing intention to use fear of technology negatively predicted acceptance
(Złotowski et al., 2015)	2015	Japan	Mixed method	58	18–36	Geminoid HI-2 & Robovie R2	Voice-adaptation (pitch entrainment), social dialogue cues, and rapport-building speech	Rapport & and social presence scales, persistence (reteaching rate), learning gains	Frontiers in Psychology	Empirical	Voice-adaptive robots increased social presence and rapport. Females showed more persistence. Learning gains were unchanged, but rapport boosted motivation. Touch and speech cues reduced awkwardness.
(Zsiga et al., 2018)	2018	Hungary	Mixed method	8	70-83	Kompaï	Long-term home use, natural language interaction, multi-modal interface	5-point Likert scale on usefulness, reliability, satisfaction; robot usage logs; CRFs	Assistive Technology (Taylor & Francis)	Empirical	Gradual introduction reduced fear; the step-by-step learning approach enhanced acceptance.

Appendix C

Table 2: Full mapping of Fear Types × Mitigation Strategies (corresponding to Figure 3). Each cell lists the specific studies (Author, Year) that contribute to the count shown in the heatmap. Cells marked with (—) indicate no studies were identified for that combination. Some studies appear in multiple cells where they addressed more than one mitigation strategy.

Fear Types	Personalization	Gradual Exposure	User Education	Interface Simplification	Transparent Design	Social Framing
**Uncanny Valley**	Appel et al. (2019); Berns & Ashok (2024); Mishra et al. (2022); Strutz et al. (2024); Dosso et al. (2023); Yam et al. (2023)	Baisch et al. (2017); Ostrowski et al. (2019); Rigaud et al. (2024)	Dosso et al. (2023); Berns & Ashok (2024); Yamaguchi (2025); Appel et al. (2019); Mishra et al. (2022); Złotowski et al. (2015); Strutz et al. (2024)	Appel et al. (2019); Dosso et al. (2023); Mishra et al. (2022); Yamaguchi (2025)	Appel et al. (2019); Berns & Ashok (2024); Mishra et al. (2022); Strutz et al. (2024); Złotowski et al. (2015); Yamaguchi (2025)	Appel et al. (2019); Berns & Ashok (2024); Mishra et al. (2022); Strutz et al. (2024); Dosso et al. (2023); Yam et al. (2023); Złotowski et al. (2015); Yamaguchi (2025); Zafrani et al. (2023)
**Privacy & Autonomy**	Coco et al. (2018); Rantanen et al. (2018)	Baisch et al. (2017); Carros et al. (2020); Cavallo et al. (2018); Rigaud et al. (2024); Søraa et al. (2022); Yam et al. (2023)	Coco et al. (2018); Rantanen et al. (2018); Rigaud et al. (2024); Søraa et al. (2022); Zsiga et al. (2018); Zafrani (2022); Yam et al. (2023)	Coco et al. (2018); Rantanen et al. (2018); Ostrowski et al. (2019); Yam et al. (2023)	Coco et al. (2018); Rantanen et al. (2018); Rigaud et al. (2024); Søraa et al. (2022); Zsiga et al. (2018)	Coco et al. (2018); Rantanen et al. (2018); Rigaud et al. (2024); Søraa et al. (2022); Zsiga et al. (2018); Zafrani (2022); Yam et al. (2023)
**Trust Issues**	Appel et al. (2019); Giorgi et al. (2022); Strutz et al. (2024); Dosso et al. (2023); Nault et al. (2024); Olatunji et al. (2025); Ostrowski et al. (2019)	Baisch et al. (2017); Carros et al. (2020)	Giorgi et al. (2022); Dosso et al. (2023); Ostrowski et al. (2019); Strutz et al. (2024); Nault et al. (2024)	Giorgi et al. (2022); Dosso et al. (2023); Zsiga et al. (2018); Coco et al. (2018)	Giorgi et al. (2022)	Giorgi et al. (2022); Strutz et al. (2024); Dosso et al. (2023); Ostrowski et al. (2019); Nault et al. (2024); Zsiga et al. (2018); Zafrani et al. (2023)
**Fear of Dependence**	Baisch et al. (2017); Dosso et al. (2023); Ostrowski et al. (2019); Ostrowski et al. (2024); Tobis et al. (2022)	Dosso et al. (2023)	Baisch et al. (2017); Dosso et al. (2023); Ostrowski et al. (2019); Olatunji et al. (2025)	Ostrowski et al. (2019)	Baisch et al. (2017); Dosso et al. (2023); Ostrowski et al. (2019); Ostrowski et al. (2024); Tobis et al. (2022); Wu et al. (2014); Zsiga et al. (2018); Rigaud et al. (2024); Zafrani et al. (2023)	Baisch et al. (2017); Dosso et al. (2023); Ostrowski et al. (2019); Ostrowski et al. (2024); Tobis et al. (2022)
**Ethical Concerns**	Jung et al. (2017); Moyle et al. (2019); Coco et al. (2018); Søraa et al. (2022); Rigaud et al. (2024); Zafrani & Nimrod (2018); Vozna & Costantini (2025); Vandemeulebroucke et al. (2019)	—	Jung et al. (2017); Moyle et al. (2019); Vandemeulebroucke et al. (2019); Sharkey & Sharkey (2012); Vozna & Costantini (2025); Coco et al. (2018); Søraa et al. (2022); Rigaud et al. (2024); Zafrani & Nimrod (2018)	Vandemeulebroucke et al. (2019); Rigaud et al. (2024)	Coco et al. (2018); Vandemeulebroucke et al. (2019); Rigaud et al. (2024); Vozna & Costantini (2025); Moyle et al. (2019); Jung et al. (2017)	Vandemeulebroucke et al. (2019); Coco et al. (2018); Rigaud et al. (2024)
**Usability Challenges**	Carros et al. (2020); Cavallo et al. (2018); Gasteiger et al. (2025); Nault et al. (2024); Olatunji et al. (2025); Strutz et al. (2024); Wu et al. (2014)	Baisch et al. (2017); Olatunji et al. (2025)	Cavallo et al. (2018); Strutz et al. (2024); Ostrowski et al. (2019); Nault et al. (2024)	Ostrowski et al. (2019); Nault et al. (2024); Cavallo et al. (2018); Gasteiger et al. (2025)	Cavallo et al. (2018); Gasteiger et al. (2025); Nault et al. (2024); Olatunji et al. (2025); Strutz et al. (2024); Wu et al. (2014)	Carros et al. (2020); Cavallo et al. (2018); Nault et al. (2024); Strutz et al. (2024)
**Unfamiliarity**	Baisch et al. (2017); Carros et al. (2020); Cavallo et al. (2018); Gasteiger et al. (2025); Nault et al. (2024); Olatunji et al. (2025); Ostrowski et al. (2019); Strutz et al. (2024)	Baisch et al. (2017); Carros et al. (2020); Olatunji et al. (2025); Yam et al. (2023); Dosso et al. (2023); Zafrani et al. (2023)	Cavallo et al. (2018); Dosso et al. (2023)	Cavallo et al. (2018); Yam et al. (2023); Ostrowski et al. (2019)	Baisch et al. (2017); Carros et al. (2020); Cavallo et al. (2018); Gasteiger et al. (2025); Nault et al. (2024); Olatunji et al. (2025); Ostrowski et al. (2019); Strutz et al. (2024)	Carros et al. (2020); Cavallo et al. (2018)

Appendix D

Table 3: Full mapping of Robot Functional Category × Mitigation Strategies (corresponding to Figure 9). Each cell lists the specific studies (Author, Year) that contribute to the count shown in the heatmap. Cells marked with (—) indicate no studies were identified for that combination. Some studies appear in multiple cells where they addressed more than one mitigation strategy.

Robot Functional Category	User-Centered Design	Gradual Exposure	Emotional Regulation	Privacy & Transparency	Adaptive Interfaces	Cultural Tailoring	Personalization	Context Responsiveness
**Assistance-oriented**	Baisch et al. (2017); Carros et al. (2020); Cavallo et al. (2018); Gasteiger et al. (2025); Ostrowski et al. (2019)	Baisch et al. (2017); Ostrowski et al. (2019)	Dosso et al. (2023); Ostrowski et al. (2019); Tobis et al. (2022)	Coco et al. (2018); Rantanen et al. (2018); Rigaud et al. (2024)	Ostrowski et al. (2019)	—	Rigaud et al. (2024)	Coco et al. (2018); Ostrowski et al. (2019)
**Therapeutic-focused**	Cavallo et al. (2018); Moyle et al. (2019)	Baisch et al. (2017)	Dosso et al. (2023); Vandemeulebroucke et al. (2019)	Coco et al. (2018)	—	—	—	Rigaud et al. (2024); Søraa et al. (2022); Zafrani et al. (2023)
**Social engagement**	Appel et al. (2019); Carros et al. (2020); Giorgi et al. (2022); Nault et al. (2024); Ostrowski et al. (2024); Strutz et al. (2024); Yam et al. (2023); Zafrani et al. (2023)	Ostrowski et al. (2019); Dosso et al. (2023)	Appel et al. (2019); Dosso et al. (2023); Yam et al. (2023); Strutz et al. (2024); Berns & Ashok (2024); Gasteiger et al. (2025)	Coco et al. (2018); Rantanen et al. (2018); Yam et al. (2023)	Carros et al. (2020); Cavallo et al. (2018); Ostrowski et al. (2019)	Yamaguchi (2025)	Appel et al. (2019); Yam et al. (2023)	Giorgi et al. (2022); Ostrowski et al. (2024); Zafrani & Nimrod (2018); Rigaud et al. (2024)
**Remote presence/surveillance**	Coco et al. (2018); Rantanen et al. (2018); Rigaud et al. (2024)	—	Ostrowski et al. (2019)	Coco et al. (2018); Rantanen et al. (2018); Rigaud et al. (2024); Søraa et al. (2022); Zsiga et al. (2018); Zafrani et al. (2023)	—	—	—	Coco et al. (2018); Rigaud et al. (2024)
**Integrated multi-function**	Appel et al. (2019); Baisch et al. (2017); Cavallo et al. (2018); Nault et al. (2024); Ostrowski et al. (2024); Strutz et al. (2024); Wu et al. (2014)	—	Dosso et al. (2023); Ostrowski et al. (2019)	Coco et al. (2018); Rantanen et al. (2018); Rigaud et al. (2024); Søraa et al. (2022)	Ostrowski et al. (2019)	Vandemeulebroucke et al. (2019)	Ostrowski et al. (2024)	Dosso et al. (2023); Zafrani et al. (2023); Ostrowski et al. (2019)

Appendix E

Table 4: Full mapping of Fear Types × Robot Functional Categories (corresponding to Figure 10). Each cell lists the specific studies (Author, Year) that contribute to the counts shown in the heatmap. Cells marked with (—) indicate no studies were identified for that combination. Some studies appear in multiple cells where they addressed more than one robot category.

Fear Type	Assistance-oriented	Therapeutic-focused	Social engagement	Remote presence / surveillance	Integrated multi-function
**Uncanny Valley**	—	Vandemeulebroucke et al. (2019)	Appel et al. (2019); Mishra et al. (2022); Strutz et al. (2024)	—	—

**Privacy & Autonomy**	Coco et al. (2018)	—	Carros et al. (2020); Søraa et al. (2022); Yam et al. (2023); Zsiga et al. (2018)	Coco et al. (2018); Rantanen et al. (2018); Rigaud et al. (2024)	Zafrani & Nimrod (2018); Zafrani et al. (2023); Dosso et al. (2023); Nault et al. (2024); Olatunji et al. (2025); Wu et al. (2014)
**Trust & Reliability**	Baisch et al. (2017); Giorgi et al. (2022)	Cavallo et al. (2018)	Giorgi et al. (2022); Appel et al. (2019); Nault et al. (2024); Strutz et al. (2024); Ostrowski et al. (2019); Yam et al. (2023); Dosso et al. (2023)	Carros et al. (2020); Coco et al. (2018); Rantanen et al. (2018); Rigaud et al. (2024); Zsiga et al. (2018)	Strutz et al. (2024); Ostrowski et al. (2024); Tobis et al. (2022); Wu et al. (2014); Gasteiger et al. (2025)
**Dependence**	—	—	Baisch et al. (2017); Dosso et al. (2023)	Ostrowski et al. (2019); Ostrowski et al. (2024)	—
**Emotional / Ethical**	Coco et al. (2018)	Vandemeulebroucke et al. (2019); Moyle et al. (2019)	Jung et al. (2017); Søraa et al. (2022); Zafrani & Nimrod (2018); Vozna & Costantini (2025)	Rigaud et al. (2024); Sharkey & Sharkey (2012)	Dosso et al. (2023); Nault et al. (2024); Olatunji et al. (2025); Yam et al. (2023); Wu et al. (2014)
**Usability**	Cavallo et al. (2018)	Carros et al. (2020)	Nault et al. (2024)	Olatunji et al. (2025)	Gasteiger et al. (2025)
**Lack of Exposure**	—	Baisch et al. (2017)	Carros et al. (2020); Ostrowski et al. (2019); Zafrani et al. (2023)	—	Cavallo et al. (2018); Gasteiger et al. (2025)
